# Supplementary material for: Socioeconomic differences in the impact of prices and taxes on tobacco use in low- and middle-income countries–A systematic review
Source: PLOS Glob Public Health. 2023 Sep 27;3(9):e0002342. doi: 10.1371/journal.pgph.0002342 (PMC10529577; doi:10.1371/journal.pgph.0002342)
Supplement: S1 Appendix — (PDF) [file pgph.0002342.s002.pdf]

S1 Appendix. Studies from low- and-middle-income countries included in the International Agency for Research on Cancer's report on the effectiveness of price and tax policies for tobacco control (IARC, 2011) — Summary of key results

**Authors/year; journal country**	**Results**
**Africa: Northern Africa** (no. of studies: 1)	
Nassar, 2003; World Bank Working Paper<br>– Egypt	Total own-price elasticity, tobacco*:<br>- quartile 1 (low): -0.36<br>- quartile 2: -0.39<br>- quartile 3: -0.41<br>- quartile 4: -0.49<br>Total own-price elasticity, tobacco*:<br>- education 1 (low): -0.44<br>- education 2: -0.44<br>- education 3: -0.41<br>- education 4: -0.44<br>* measures of uncertainty not reported; 1999/2000.<br>Source: Tables 25, 26
**Africa: Sub-Saharan Africa** (no. of studies: 1)	
van Walbeek, 2002; South African Journal of Economics<br>– South Africa	Total own-price elasticity, cigarettes*:<br>- quartile 1 (low): -1.39<br>- quartile 2: -1.13<br>- quartile 3: -1.08<br>- quartile 4: -0.81<br>* measures of uncertainty/significance level not reported.<br>Source: Table 7
**Americas: Caribbean** (no. of studies: 0)	
**Americas: Latin America** (no. of studies: 0)	
**Asia: Central Asia** (no. of studies: 0)	
**Asia: Eastern Asia** (no. of studies: 3)	
Lance, Akin et al., 2004; Journal of Health Economics<br>– China	Total own-price elasticity, cigarettes*:<br>Pooled cross-sectional specification:<br>- wealth 1 (low): -0.14<br>- wealth 2: 0.04<br>- wealth 3: -0.12<br>- wealth 4: -0.12<br>Community-level fixed effects specification:<br>– all: -0.01<br>- wealth 1 (low): 0.04<br>- wealth 2: 0.10<br>- wealth 3: -0.05<br>- wealth 4: -0.18<br>* measures of uncertainty/ significance level not reported; prices collectively significant in participation part, but not consumption.<br>Source: Table 3

Authors/year; journal country	Results
Mao, Yang et al., 2003; Soft Science of Health<br>– China	Total own-price elasticity, cigarettes*:<br>– all: -0.51<br>- poor: -1.91<br>- low-income: -0.77<br>- high-income: -0.51<br>* measures of uncertainty/significance level not reported; poor, household income < 200 RMB/month; low income, household income < 500 RMB/month; high income, household income > 500 RMB/month<br>Source: IARC (2011) Table 5.1
Mao, Sung et al., 2008; book chapter<br>– China	Participation own-price elasticity, cigarettes:<br>– all: -0.06 (95%Cl -0.12, -0.01)<br>- poor: -0.48 (95%Cl -0.60, -0.36)<br>- low-income: -0.20 (95%Cl -0.31, -0.09)<br>- middle-income: 0.09 (95%Cl 0.01, 0.18)<br>- high-income: 0.34 (95%Cl 0.17, 0.51)<br>Consumption own-price elasticity, cigarettes:<br>– all: -0.09 (95%Cl -0.13, -0.05)<br>- poor: -0.11 (95%Cl -0.191 -0.03)<br>- low-income: -0.04 (95%Cl -0.11, 0.04)<br>- middle-income: -0.11 (95%Cl -0.17, -0.05)<br>- high-income: -0.08 (95%Cl -0.22, 0.05)<br>Total own-price elasticity, cigarettes:<br>– all: -0.15 (95%Cl -0.21, -0.09)<br>- poor: -0.59 (95%Cl -0.74, -0.44)<br>- low-income: -0.23 (95%Cl -0.37, -0.10)<br>- middle-income: -0.02 (95%Cl -0.12, 0.09)<br>- high-income: 0.26 (95%Cl 0.04, 0.47)<br>Source: Table 4
**Asia: South-eastern Asia** (no. of studies: 5)	
Adioetomo, Djutaharta, Hendratno, 2005; World Bank Working Paper<br>– Indonesia	Participation own-price elasticity, cigarettes*:<br>- low-income: -0.03 (P > 0.05)<br>- middle: 0.09 (P > 0.05)<br>- high: 0.20 (P > 0.05)<br>Consumption own-price elasticity, cigarettes*:<br>- low-income: -0.66 (P < 0.01)<br>- middle: -0.37 (P < 0.01)<br>- high: -0.41 (P < 0.01)<br>Total own-price elasticity, cigarettes*:<br>- low-income: -0.67<br>- middle: -0.33<br>- high: -0.31<br>* measures of uncertainty not reported.<br>Source: Table 9

Authors/year; journal country	Results
Kyaing, 2003; World Bank Working Paper<br>– Myanmar	Participation own-price elasticity, tobacco*:<br>- quintile 1 (low): -1.09<br>- quintile 2: -1.25<br>- quintile 3: -1.41<br>- quintile 4: -1.38<br>- quintile 5: -1.24<br>Consumption own-price elasticity, tobacco:<br>- quintile 1 (low): -0.42 (95%CI -0.51, -0.33)<br>- quintile 2: -0.31 (95%CI -0.39, -0.23)<br>- quintile 3: -0.34 (95%CI -0.46, -0.23)<br>- quintile 4: -0.36 (95%CI -0.46, -0.26)<br>- quintile 5: -0.24 (95%CI -0.34, -0.14)<br>Total own-price elasticity, tobacco*:<br>- quintile 1 (low): -1.06<br>- quintile 2: -1.56<br>- quintile 3: -1.75<br>- quintile 4: -1.73<br>- quintile 5: -1.48<br>* measures of uncertainty not reported; tobacco = cigarettes, cheroots, phet kyan.<br>Source: Table 4.11
Kyaing, Perucic, Rahman, 2005; World Bank Working Paper<br>– Myanmar	Consumption own-price elasticity, cheroots:<br>- quartile 1 (low): -0.50 (95% CI -0.66, -0.34)<br>- quartile 2: -0.44 (95% CI -0.59, -0.29)<br>- quartile 3: -0.22 (95% CI -0.37, -0.07)<br>- quartile 4: -0.32 (95% CI -0.59, -0.05)<br>Consumption own-price elasticity, cigarettes:<br>- quartile 1 (low): -0.15 (95% CI -0.48, 0.18)<br>- quartile 2: -0.37 (95% CI -0.61, -0.13)<br>- quartile 3: -0.25 (95% CI -0.44, -0.06)<br>- quartile 4: -0.14 (95% CI -0.39, 0.11)<br>Consumption own-price elasticity, cheroots/cigarettes:<br>- quartile 1 (low): -0.29 (95% CI -0.54, -0.04)<br>- quartile 2: -0.19 (95% CI -0.54, -0.16)<br>- quartile 3: -0.10 (95% CI -0.37, 0.17)<br>- quartile 4: -0.27 (95% CI -0.72, 0.18)<br>Source: Tables 17, 18, 19
Sarntisart, 2003; World Bank Working Paper<br>– Thailand	Total own-price elasticity, cigarettes*:<br>Urban:<br>- quintile 1 (low): -1.00<br>- quintile 2: -0.36<br>- quintile 3: -0.13<br>- quintile 4: -0.10<br>- quintile 5: -0.04<br>Rural:<br>- quintile 1 (low): -0.49<br>- quintile 2: -0.05<br>- quintile 3: -0.03<br>- quintile 4: -0.15<br>- quintile 5: -0.07<br>* measures of uncertainty/significance level not reported.<br>Source: Table 4.3

Authors/year; journal country	Results
Van Kinh, Ross et al., 2006; Health Res Policy Syst<br>– Vietnam	Participation own-price elasticity, cigarettes*:<br>- quintiles 1, 2 (low): -1.16 (P < 0.10)<br>- quintiles 4, 5 (high): -0.75 (P < 0.10)<br>Consumption own-price elasticity, cigarettes*:<br>- quintiles 1, 2 (low): -0.61 (P < 0.10)<br>- quintiles 4, 5 (high): -0.42 (P < 0.10)<br>* measures of uncertainty not reported; male only; VINATABA prices.<br>Source: Text, Tables 3, 4.
**Asia: Southern Asia** (no. of studies: 3)	
Karki, Pant, Pande, 2003; World Bank Working Paper<br>– Nepal	Participation own-price elasticity, cigarettes/bidis*:<br>- quintile 1 (low): -0.31 (P < 0.01)<br>- quintile 2: -0.26 (P < 0.01)<br>- quintile 3: -0.35 (P < 0.01)<br>- quintile 4: -0.35 (P < 0.01)<br>- quintile 5: -0.31 (P < 0.01)<br>Consumption own-price elasticity, cigarettes/bidis*:<br>- quintile 1 (low): -0.48 (95%CI -0.66, -0.30)<br>- quintile 2: -0.61 (95%CI -0.80, -0.42)<br>- quintile 3: -0.48 (95%CI -0.72, -0.24)<br>- quintile 4: -0.28 (95%CI -0.48, -0.08)<br>- quintile 5: -0.29 (95%CI -0.50, -0.08)<br>Total own-price elasticity, cigarettes/bidis*:<br>- quintile 1 (low): -0.79<br>- quintile 2: -0.86<br>- quintile 3: -0.83<br>- quintile 4: -0.63<br>- quintile 5: -0.60<br>* measures of uncertainty not reported.<br>Source: Table 4.10
Arunatilake, 2002; Sri Lanka Economic Journal<br>– Sri Lanka	Participation own-price elasticity, cigarettes*:<br>- quintile 1 (low): -0.17 (P > 0.1)<br>- quintile 2: 0.17 (P > 0.1)<br>- quintile 3: 0.21 (P < 0.1)<br>- quintile 4: 0.01 (P > 0.1)<br>- quintile 5: 0.34 (P < 0.05)<br>Consumption own-price elasticity, cigarettes*:<br>- quintile 1 (low): -0.52 (P < 0.01)<br>- quintile 2: -0.67 (P < 0.01)<br>- quintile 3: -0.74 (P < 0.01)<br>- quintile 4: -0.69 (P < 0.01)<br>- quintile 5: -0.56 (P < 0.01)<br>Total own-price elasticity, cigarettes*:<br>- quintile 1 (low): -0.64<br>- quintile 2: -0.55<br>- quintile 3: -0.60<br>- quintile 4: -0.68<br>- quintile 5: -0.29<br>* measures of uncertainty/significance level not reported.<br>Source: Table 4.

Authors/year; journal country	Results
Arunatilake, Opatha, 2003; World Bank Working Paper<br>– Sri Lanka	Participation own-price elasticity, cigarettes*:<br>- quintile 1 (low): -0.17 (P > 0.10)<br>- quintile 2: 0.17 (P > 0.10)<br>- quintile 3: 0.21 (P < 0.1)<br>- quintile 4: 0.01 (P > 0.10)<br>- quintile 5: 0.34 (P < 0.05)<br>Consumption own-price elasticity, cigarettes*:<br>- quintile 1 (low): -0.52 (P < 0.01)<br>- quintile 2: -0.67 (P < 0.01)<br>- quintile 3: -0.74 (P < 0.01)<br>- quintile 4: -0.69 (P < 0.01)<br>- quintile 5: -0.56 (P < 0.01)<br>* measures of uncertainty not reported.<br>Source: Table 26
**Asia: Western Asia** (no. of studies: 1)	
Onder, 2002; World Bank Working Paper<br>- Turkey	Participation own-price elasticity, cigarettes*:<br>- quintile 1 (low): -0.12 (P < 0.01)<br>- quintile 2: -0.32 (P < 0.01)<br>- quintile 3: -0.11 (P < 0.01)<br>- quintile 4: -0.02 (P < 0.01)<br>- quintile 5: 0.15 (P < 0.01)<br>Consumption own-price elasticity, cigarettes*:<br>- quintile 1 (low): -0.34 (P < 0.01)<br>- quintile 2: -0.58 (P < 0.01)<br>- quintile 3: -0.45 (P < 0.01)<br>- quintile 4: -0.41 (P < 0.01)<br>- quintile 5: -0.30 (P < 0.01)<br>Total own-price elasticity, cigarettes*:<br>- quintile 1 (low): -0.47 (P < 0.01)<br>- quintile 2: -0.90 (P < 0.01)<br>- quintile 3: -0.56 (P < 0.01)<br>- quintile 4: -0.43 (P < 0.01)<br>- quintile 5: -0.16 (P < 0.01)<br>* measures of uncertainty not reported<br>Source: Table 4.11
**Europe: Eastern Europe** (no. of studies: 3)	
Sayginsoy, Yurekli, de Beyer, 2002; World Bank Working Paper<br>– Bulgaria	Consumption own-price elasticity, cigarettes*:<br>All: -0.8 (P < 0.01)<br>- low- and low-middle income household: -1.33 (P < 0.01)<br>- high-middle income household: -1.02 (P < 0.01)<br>- high-income household: -0.52 (P < 0.01)<br>* measures of uncertainty not reported.<br>Source: Table 3

Authors/year; journal country	Results
Lance, Akin et al., 2004; Journal of Health Economics<br>– Russia	Total own-price elasticity, cigarettes*:<br>Pooled cross-sectional specification:<br>- wealth 1 (low): -0.11<br>- wealth 2: -0.12<br>- wealth 3: -0.12<br>- wealth 4: -0.18<br>Community-level fixed effects specification:<br>- wealth 1 (low): 0.01<br>- wealth 2: 0.02<br>- wealth 3: -0.07<br>- wealth 4: -0.20<br>* measures of uncertainty/significance level not reported; prices not collectively significant in participation part, but not consumption.<br>Source: Table 3
Krasovsky, Andreeva et al., 2002; report<br>– Ukraine	Participation own-price elasticity, cigarettes*:<br>– all: -0.47<br>- low-income: -0.27<br>- high-income: -1.10<br>Consumption own-price elasticity, cigarettes*:<br>Age: 14-17; 18-28; > 28 years<br>- low-income: -0.65; -0.37;-0.28<br>- middle-income: -0.70; -0.42; -0.33<br>- high-income: -0.52; -0.24; -0.15<br>* measures of uncertainty/significance level not reported;<br>Source: IARC (2011) Table 5.1
**Oceania** (no. of studies: 0)	
Note: Geographical regions are based on continental regions; which are further subdivided into sub-regions (United Nations Statistics Division, https://unstats.un.org/unsd/methodology/m49/).	

International Agency for Research on Cancer (2011). IARC Handbooks of Cancer Prevention: Tobacco Control. Volume 14. Effectiveness of Price and Tax Policies for Tobacco Control. Lyon: International Agency for Research on Cancer.
