# Supplementary material for: Socioeconomic differences in the impact of prices and taxes on tobacco use in low- and middle-income countries–A systematic review
Source: PLOS Glob Public Health. 2023 Sep 27;3(9):e0002342. doi: 10.1371/journal.pgph.0002342 (PMC10529577; doi:10.1371/journal.pgph.0002342)
Supplement: S2 Appendix — (PDF) [file pgph.0002342.s003.pdf]

S2 Appendix. Search strategy

MEDLINE

(pric*[Title/Abstract] OR "Taxes"[MeSH] OR tax*[Title/Abstract]) AND (smok*[Title/Abstract] OR tobacco*[Title/Abstract] OR "Tobacco"[MeSH] OR "Smoking"[MeSH]) AND ([MeSH]Africa OR Europe OR Pacific OR Australasia OR Asia OR "Latin America"OR Carribean" OR "Middle East") OR ([Abstract/Title] Algeria OR Angola OR Benin OR Botswana OR Burkina Faso OR Burundi OR Cameroon OR Cape Verde OR Cabo verde OR Central African Republic OR Chad OR Comoros OR Congo OR "Côte d'Ivoire" OR Djibouti OR Egypt OR Eritrea OR Eswatini OR Ethiopia OR Gabon OR Gambia OR Ghana OR Guinea OR Kenya OR Lesotho OR Liberia OR Libya OR Madagascar OR Mauritius OR Malawi OR Mali OR Mauritania OR Morocco OR Mozambique OR Namibia OR Niger OR Nigeria OR Rwanda OR "São Tomé and Principe" OR Senegal OR Seychelles OR Sierra Leone OR Somalia OR South africa OR Sudan OR Swaziland OR Tanzania OR Togo OR Tunisia OR Uganda OR Zambia OR Zimbabwe OR Kiribati OR Micronesia OR Vanautu OR Fiji OR Marshall Islands OR Nauru OR Samoa OR Tuvalu OR Indonesia OR Kyrgyz-Republic OR Laos OR Mongolia OR Myanmar OR Burma OR Philippines OR Timor-leste OR North-Korea OR "Democratic People's Republic of Korea" OR Uzbekistan OR Vietnam OR China OR Malaysia OR Moldova OR Morocco OR Ukraine OR Albania OR Belarus OR Kosovo OR North-Macedonia OR Romania OR Russian Federation OR Russia OR Serbia OR "Isle of Man" OR Georgia OR Poland OR Czech OR Czech Republic OR Greece OR Portugal OR Hungary OR Bulgaria OR Slovakia OR slovak republic OR Slovenia OR Croatia OR Bosnia OR Herzegovina OR Luthuania OR Latvia OR Estonia OR Montenegro OR Malta OR Armenia OR Azerbaijan OR Antigua OR Barbuda OR Dominican OR Nicaragua OR Argentina OR Ecuador OR Panama OR Belize OR Salvador OR Paraguay OR Bolivia OR Grenada OR Peru OR Brazil OR Guatemala OR Kitts OR Nevis OR Chile OR Guyana OR Lucia OR Colombia OR Haiti OR Vincent OR Costa Rica OR Honduras OR Suriname OR Cuba OR Jamaica OR Uruguay OR Dominica OR Mexico OR Venezuela OR Bahamas OR Barbados OR Trinidad OR Tobago OR Puerto Rico OR Guadeloupe OR Martinique OR Curacao OR Aruba OR Grenadines OR Cayman Islands OR sint maarten OR saint martin OR turks OR caicos OR british virgin islands OR caribbean netherlands OR Anguilla OR saint barthelemy OR Montserrat OR Syria OR Tajikistan OR Iran OR Iraq OR Jordan OR Kazakhstan OR Lebanon OR Turkey OR Turkeministan OR Uzbekistan OR Israel OR Yemen OR Saudi-Arabia OR Oman OR Cyprus OR Bahrain OR Palestine OR India OR Pakistan OR Bangladesh OR Maldives OR Nepal OR Afghanistan OR Sri Lanka OR Bhutan)). Limit 2010-current

EconLit

(TI(tobacco* OR smoke* OR cigar*) OR AB(tobacco* OR smoke* OR cigar*)) AND (TI(tax* OR price*) OR AB(tax* OR price*)) AND (su.Exact(Africa OR Europe OR Pacific OR Australasia OR Asia OR Latin America OR Carribean" OR Middle East) OR (TI(Africa OR Europe OR Pacific OR Australasia OR Asia OR Latin America OR Carribean OR Middle East) OR AB(Africa OR Europe OR Pacific OR Australasia OR Asia OR Latin America OR Carribean OR Middle East)) OR Algeria OR Angola OR Benin OR Botswana OR "Burkina Faso" OR Burundi OR Cameroon OR "Cape Verde" OR "Cabo verde" OR "Central African Republic" OR Chad OR Comoros OR Congo OR Côte d'Ivoire OR Djibouti OR Egypt OR Eritrea OR Eswatini OR Ethiopia OR Gabon OR Gambia OR Ghana OR Guinea OR Kenya OR Lesotho OR Liberia OR Libya OR Madagascar OR Mauritius OR Malawi OR Mali OR Mauritania OR Morocco OR Mozambique OR Namibia OR Niger OR Nigeria OR Rwanda OR São Tomé and Principe OR Senegal OR Seychelles OR Sierra Leone OR Somalia OR "South africa" OR Sudan OR Swaziland OR Tanzania OR Togo OR Tunisia OR Uganda OR Zambia OR Zimbabwe OR Kiribati OR Micronesia OR Vanautu OR Fiji OR Marshall Islands OR Nauru OR Samoa OR Tuvalu OR Indonesia OR Kyrgyz-Republic OR Laos OR Mongolia OR Myanmar OR Burma OR Philippines OR Timor-leste OR North-Korea OR "Democratic People's Republic of Korea" OR Uzbekistan OR Vietnam OR China OR Malaysia OR Moldova OR Morocco OR Ukraine OR Albania OR Belarus OR Kosovo OR North-Macedonia OR Romania OR Russian Federation OR Russia OR Serbia OR "Isle of Man" OR Georgia OR Poland OR Czech OR Czech Republic OR Greece OR Portugal OR Hungary OR Bulgaria OR Slovakia OR slovak republic OR Slovenia OR Croatia OR Bosnia OR Herzegovina OR Luthuania OR Latvia OR Estonia OR Montenegro OR Malta OR Armenia OR Azerbaijan OR Antigua OR Barbuda OR Dominican OR Nicaragua OR Argentina OR Ecuador OR Panama OR Belize OR Salvador OR Paraguay OR Bolivia OR Grenada OR Peru OR Brazil OR Guatemala OR Kitts OR Nevis OR Chile OR Guyana OR Lucia OR Colombia OR Haiti OR Vincent OR Costa Rica OR Honduras OR Suriname OR Cuba OR Jamaica OR Uruguay OR Dominica OR Mexico OR Venezuela OR Bahamas OR Barbados OR Trinidad OR Tobago OR Puerto Rico OR Guadeloupe OR Martinique OR Curacao OR Aruba OR Grenadines OR Cayman Islands OR sint maarten OR saint martin OR turks OR caicos OR british virgin islands OR caribbean netherlands OR Anguilla OR saint barthelemy OR Montserrat OR Syria OR Tajikistan OR Iran OR Iraq OR Jordan OR Kazakhstan OR Lebanon OR Turkey OR Turkeministan OR Uzbekistan OR Israel OR Yemen OR Saudi-Arabia OR Oman OR Cyprus OR Bahrain OR Palestine OR India OR Pakistan OR Bangladesh OR Maldives OR Nepal OR Afghanistan OR Sri Lanka OR Bhutan
