# Supplementary material for: Socioeconomic differences in the impact of prices and taxes on tobacco use in low- and middle-income countries–A systematic review
Source: PLOS Glob Public Health. 2023 Sep 27;3(9):e0002342. doi: 10.1371/journal.pgph.0002342 (PMC10529577; doi:10.1371/journal.pgph.0002342)
Supplement: S3 Appendix — (PDF) [file pgph.0002342.s004.pdf]

S3 Appendix. Study characteristics

— Africa: Sub-Saharan Africa

Dauchy E, Ross H. The effect of price and tax policies on the decision to smoke among men in Kenya. Addiction 2019;114(7):1249-63. doi: 10.1111/add.14623

Country/Journal	- Kenya<br>- Addiction
Data	- Type: retrospective constructed from cross-sectional data (2014);<br>- Sample size: 2079 male survey respondents; 619 male ever-smokers; 1925 at risk of initiation; 478 ever-smokers at risk of cessation;<br>- Population: representativeness unclear<br>- Missing data: unclear;<br>- Source: Global Adult Tobacco Survey.
Methods	Pooled linear probability model with propensity score matching (initiation only):<br>- Baseline: pooled OLS (prices in ln);<br>- First step: probit (full-sample); estimated likelihood of participation based on time-invariant characteristics;<br>- Second step: reweighed data with variance-covariance matrix obtained from first step; estimated impact of price on initiation on subsample narrowed to ever-smokers and their nearest matches;<br>- Studied likelihood of initiation and cessation for individual (i) in period (t) as a function of ln(cigarette prices);<br>Price elasticities based on delta method<br>Functional form of duration/time dependency not clearly described; age, age squared included as covariates.
Dependent variables	- smoking initiation; no clear definition provided;<br>- age at first risk of starting: unclear<br>- cessation; no clear definition provided;
Price/tax variable(s)	Real prices of Crown Bird (1960-1967) and Sportman (1968-2014) cigarettes;<br>Deflator: CPI all-items<br>Source: Kenya National Statistics Bureau
Covariates	Age, urban/rural, marital status, education, occupation, wealth.
Misspecification tests	None reported.
Sensitivity analyses	- Propensity score matching; narrowed group of never-smokers to those who best matched ever-smokers in terms of observed covariates; less likely to be subject to endogeneity<br>- Split duration model; hazard functions (logistic distribution).
Results	Initiation own-price elasticity, cigarettes:<br>– all, males: -0.03 (95%CI -0.07, -0.00)<br>– lowest wealth tercile, males: -0.02 (95%CI -0.08, 0.04)<br>Cessation own-price elasticity, cigarettes:<br>– all, males: 0.03 (95%CI -0.26, 0.32)<br>– lowest wealth tercile, males: 0.16 (95%CI -0.75, 0.43)<br>*Summary of findings*: no evidence of any statistically or economically significant socioeconomic differences in price responsiveness for cigarette smoking initiation or cessation.<br><br>Source: Tables 5, 7.

Limitations	- unclear description of dependent variables;<br>- no testing for misspecification;<br>- functional form of duration/time dependency not clearly reported;<br>- unclear how prices were matched to retrospective individual level data;<br>- imprecision: uncertainty intervals very wide;<br>- no formal assessment of socioeconomic differences in price responsiveness.<br>Risk of bias: high
Funding, competing interests reported?	Funding: International Development Research Center (IDRC);<br>Competing interests: disclosed (none reported).

Kidane A, Hepelwa A, Mdadila K, et al. The demand for cigarette in Tanzania: A temporal approach. Applied Econometrics 2017;45:136-43.

Country/Journal	- Tanzania<br>- Applied Econometrics
Data	- Type: repeated cross-sectional (2008, 2010, 2012)<br>- Sample size: 3265 hh (2008), 3924 hh (2010) and 5010 hh (2012)<br>- Population: hh, representative at national level<br>- Missing data: not reported<br>- Source: Tanzanian Household Budget survey (Tanzanian National Bureau of Statistics)
Methods	Two-part model:<br>- Participation: logit (prices in ln)<br>- Consumption: OLS (functional form: ln-ln)
Dependent variables	- Participation: currently smoking; 'smoking' not defined;<br>- Consumption: packs of cigarettes smoked per month, conditional on smoking
Price/tax variable(s)	Regional unit values in 2008 (total cigarette expenditure/sticks purchased); # of regions not reported; 2010, 2012 prices estimated by multiplying 2008 prices by CPI cigarette; converted to packs by taking the average quantity of three representative brands;<br>Deflator: unclear<br>Source: not reported
Covariates	Sex, residence, age, education, total annual expenditure.
Misspecification tests	None reported
Sensitivity analyses	None reported

Results	Total own-price elasticity, cigarette:<br>– 2008<br>- all: -0.7<br>- very poor: -0.3<br>- poor: -0.8<br>- middle: -1.6<br>- high: -0.3<br>– 2010<br>- all: -0.7<br>- very poor: -1.2<br>- poor: -1.5<br>- middle: 0.2<br>- high: -0.8<br>– 2012<br>- all: -1.1<br>- very poor: -2.4<br>- poor: -2.0<br>- middle: -1.0<br>- high: -0.6<br><br>Measures of uncertainty not reported; participation/consumption own-price elasticities not reported.<br><br>*Summary of findings*: it is unclear if there were any statistically or economically significant socioeconomic differences in price responsiveness.<br><br>Source: Table 4
Limittations	- unclear description of dependent variable;<br>- unclear reporting of missing data/outliers;<br>- # of clusters not reported;<br>- unclear adjustment for inflation;<br>- no testing for misspecification;<br>- no sensitivity analyses;<br>- no account for measurement error or quality;<br>- imprecision: unclear; likely very high (measures of uncertainty not reported);<br>- no formal assessment of socioeconomic differences in price responsiveness;<br>- differences in own-price elasticities across time and between expenditure groups not believable;<br>- conclusion not supported by results<br>Risk of bias: very high
Funding, competing interests reported?	Funding: US National Institutes of Health-Fogarty International Center; US National Cancer Institute;<br>Competing interests: not disclosed.

— Americas: Latin America

Guindon GE, Paraje GR, Chávez R. Prices, inflation, and smoking onset: the case of Argentina. Econ Inq 2018;56(1):424-45. doi: doi:10.1111/ecin.12490

Country/Journal	- Argentina<br>- Economic Inquiry
Data	- Type: retrospective constructed from cross-sectional data (2005, 2008, 2009, 2011);<br>- Sample size: 62,669 survey respondents to two national surveys;<br>- Population: nationally and provincially representative mostly urban surveys (16-65 and 18-65 years);<br>- Missing data: listwise deletion;<br>- Source: Encuesta Nacional Factores de Riesgo; and, Encuesta Nacional sobre Prevalencias de Consumo de Sustancias Psicoactivas.
Methods	Survival/duration analyses:<br>- discrete-time hazard models and a complementary loglog specification;<br>- functional form of baseline hazard function: cubic polynomial specification.
Dependent variables	- age at smoking onset (how old were you when you smoked for the first time?); month/year of onset randomly selected using uniform distribution; sensitivity analyses using mid-point;<br>- age at first risk of starting: 8
Price/tax variable(s)	Manufactured tobacco component (of which cigarettes represent nearly 100%) of Argentina's consumer price index (CPI) for Greater Buenos Aires (Jan 1980 - May 2008); after-tax monthly weighted average price for a pack of 20 cigarettes (> May 2008);<br>Deflator: CPI all-items Greater Buenos Aires (1980 - 2006); CPI all-items, Sante Fe province (> 2006); MIT's Billion Prices Project (> 2006);<br>Source: Instituto Nacional de Estadística y Censos (INDEC); the Ministerio de Agricultura, Ganadería y Pesca; MIT's Billion Prices Project.
Covariates	Alcohol prices; periods of hyperinflation and very high inflation; sex; household head's education level; 1986 national tobacco control policies; province-level smoke free policies; provinces; survey wave.
Misspecification tests	None reported.
Sensitivity analyses	- estimated discrete-time split population models;<br>- estimated all models with and without sampling weights;<br>- included a measure of calendar time (controlled for differing birth cohorts);<br>- estimated models using sample restricted to individuals that were between 16 and 33 years old when interviewed;<br>- re-estimated all models using annual data;<br>- alternative functional form for the baseline hazard function (dummy specification for time at risk, measured in years);<br>- estimated models assuming that individuals were first exposed to the risk of starting to smoke at age 0, 5, and 11.

Results	Initiation own-price elasticity, cigarettes:<br>- all: -0.41 (95%CI -0.60, -0.21)<br>- Mother's educational level<br>- primary or less: -0.21 (95%CI -0.55, 0.12)<br>- secondary or less: -0.55 (95%CI -0.84, -0.26)<br>- more than secondary: -0.77 (95%CI -1.10, -0.44)<br>*Summary of findings*: no evidence of any statistically significant socioeconomic differences in price responsiveness for cigarette smoking initiation. Results, if anything, suggest that lower SES individuals may have been less responsive to price.<br><br>Source: Table 3, model 2.
Limitations	- no testing for misspecification;<br>- non informative censoring among younger survey respondents;<br>- imprecision: uncertainty intervals fairly wide.<br>Risk of bias: moderate
Funding, competing interests reported?	Funding: International Development Research Center (IDRC);<br>Competing interests: disclosed (none reported).

Divino JA, Ehrl P, Candido O, et al. Extended cost-benefit analysis of tobacco taxation in Brazil. Tob Control 2021 doi: 10.1136/tobaccocontrol-2021-056806

Country/Journal	- Brazil<br>- Tobacco Control
Data	- Type: repeated cross-sectional data (2008, 2013)<br>- Sample size: not reported<br>- Population: not clearly reported<br>- Missing data: not reported;<br>- Source: 2008 National Household Sample Survey (PNAD); 2013 National Health Survey (PNS)
Methods	Two-part model:<br>- Participation: Probit;<br>- Consumption: unclear
Dependent variables	- Participation: smoker or not; 'smoker' not defined;<br>- Consumption: number of cigarettes smoked per day, conditional on smoking
Price/tax variable(s)	Mean of self-reported prices across smokers at federal state level; no. of states not reported; Brazil has 26 states (estados) and one federal district (distrito federal).<br>Deflator: unclear<br>Source: unclear
Covariates	Income quartile; age; education; years of smoking; sex; federal state fixed effects.
Misspecification tests	None
Sensitivity analyses	None

Results	Total own-price elasticity, cigarette:<br>- Income quartile 1: -0.47 (95%CI -0.68, -0.23)<br>- Income quartile 2: -0.49 (95%CI -0.69, -0.25)<br>- Income quartile 3: -0.52 (95%CI -0.71, -0.28)<br>- Income quartile 4: -0.55 (95%CI -0.75, -0.31)<br>Own-price participation and consumption elasticities not reported.<br><br>*Summary of findings*: No evidence of any statistically or economically significant socioeconomic differences in price responsiveness for cigarette smoking.<br><br>Source: Table 2
Limitations	- unclear description of dependent variables, data and methods;<br>- unclear reporting of missing data/outliers;<br>- unclear adjustment for inflation;<br>- no. of clusters not clearly reported; small no. of clusters (≤ 27) and time periods (2)<br>- no testing for misspecification;<br>- no sensitivity analysis;<br>- imprecision: uncertainty intervals fairly wide;<br>- no formal assessment of socioeconomic differences in price responsiveness.<br>Risk of bias: very high
Funding, competing interests reported?	Funding: University of Iillinois, Chicago's Institute for Health Research and Policy with Bloombert Philanthropies<br>Competing interests: disclosed (none reported)

Paraje G, Araya D, De Paz A, et al. Price and expenditure elasticity of cigarette demand in El Salvador: a household-level analysis and simulation of a tax increase. Tob Control 2021;30(4):422. doi: 10.1136/tobaccocontrol-2019-055568

Country/Journal	- El Salvador<br>- Tobacco Control
Data	- Type: cross-sectional (2005/2006);<br>- Sample size: 4332 hh<br>- Population: unclear<br>- Missing data: listwise deletion (95% response rate)<br>- Source: Encuesta de Ingresos y Gastos de Los Hogares 2005-2006
Methods	Almost Ideal Demand System, AIDS
Dependent variables	- share of the budget devoted to cigarettes.
Price/tax variable(s)	Unit values (expenditures/quantity consumed) at cluster-level (468 primary sampling units)<br>Deflator: unclear<br>Source: n/a
Covariates	No. of individuals ≥ 15 years in hh; share of women ≥ 15 years in hh; hh head education, sex, age.
Misspecification tests	None
Sensitivity analyses	None

Results	Own-price elasticities, cigarettes:<br>- All: -0.8 (95%CI -0.9, -0.6)<br>- Household total expenditures :<br>- quintiles 1,2 (low): -1.4 (95%CI -35, 32)<br>- quintile 5 (high): -0.9 (95%CI -1.3, -0.5)<br>*Summary of findings*: no evidence of any statistically significant socioeconomic difference in price responsiveness; estimates are too imprecise to assess whether difference may have been economically significant.<br><br>Source: Table 2
Limitations	- unclear adjustment for inflation;<br>- no. of clusters not reported; no. of household with positive cigarette expenditures within each cluster not clearly reported;<br>- no account for measurement error;<br>- no testing for misspecification;<br>- no sensitivity analysis;<br>- imprecision: uncertainty intervals very wide;<br>- no formal assessment of socioeconomic differences in price responsiveness.<br>Risk of bias: high
Funding, competing interests reported?	Funding: International Union Against Tuberculosis and Lung Disease; the Pan-American Health Organization;<br>Competing interests: disclosed (none reported).

de los Ríos C, Medina D, Aguilar J. Cost-benefit analysis of tobacco consumption in Peru. Documento de Trabajo, 270. Estudio Sobre Desarrollo, 43. Lima: Instituto de Estudios Peruanos, 2020.

Country/Journal	- Peru<br>- Working paper, Instituto de Estudios Peruanos
Data	- Type: cross-sectional (2009);<br>- Sample size: 35,000 hh in 385 districts<br>- Population: hh, representativeness not clearly reported;<br>- Missing data: unclear<br>- Source: Encuesta de Ingresos y Gastos de Los Hogares 2005-2006
Methods	Two-part model:<br>- Participation: Probit;<br>- Consumption: Deaton's two-equation system of budget shares and unit values
Dependent variables	- Participation: not clearly reported; likely positive household expenditures on cigarettes vs. no expenditures on cigarettes.<br>- Consumption: share of the budget devoted to cigarettes.
Price/tax variable(s)	Unit values (expenditures/quantity consumed) at cluster-level (249 districts with at least one household with positive cigarette expenditure); districts that had fewer than two observations with positive cigarette expenditures were dropped from participation part;<br>Deflator: unclear<br>Source: n/a
Covariates	Household share of/with: women/men; higher education/secondary education; highest level of education reached by any member; working age; hh head education, sex, age, working status.<br>– unclear how variables were operationalized as some categories seem to be perfectly correlated.

Misspecification tests	None
Sensitivity analyses	None
Results	Participation own-price elasticity, cigarettes:<br>- all: -0.50 (95%CI -0.90, -0.08)<br>- Household total expenditures<br>- tercile 1, low: -0.70 (95%CI -0.90, -0.08)<br>- tercile 2, mid: -0.26 (95%CI -0.66, 0.16)<br>- tercile 3, high: -0.30 (95%CI -0.61, 0.02)<br>Consumption own-price elasticity, cigarettes:<br>- all: -0.99 (95%CI -1.07, -0.91)<br>- Household total expenditures<br>- tercile 1, low: -1.02 (95%CI -1.12, -0.93)<br>- tercile 2, mid: -0.87 (95%CI -1.10, -0.63)<br>- tercile 3, high: -0.56 (95%CI -1.01, -0.10)<br>*Summary of findings*: estimates suggest that lower socioeconomic status household were more responsive to price; differences were large enough to be economically meaningful.<br>Source: Tables 5, 6, 7.
Limitations	- unclear description of dependent variables and covariates;<br>- unclear reporting of missing data/outliers;<br>- unclear adjustment for inflation;<br>- no. of household with positive cigarette expenditures within each cluster not clearly reported; clusters (districts) with only 1 household with positive cigarette expenditures included in consumption part; clusters with 2 or more included in both participation and consumption parts;<br>- no testing for misspecification;<br>- no sensitivity analysis;<br>- imprecision: uncertainty intervals very wide;<br>- no formal assessment of socioeconomic differences in price responsiveness.<br>Risk of bias: high
Funding, competing interests reported?	Funding: not disclosed<br>Competing interests: not disclosed

Gallego JM, Otalvaro-Ramirez S, Rodriguez-Lesmes PA. Price smoking participation elasticity in Colombia: estimates by age and socioeconomic level. Tob Control 2020 doi: 10.1136/tobaccocontrol-2019-055186

Country/Journal	- Colombia<br>- Tobacco Control
Data	- Type: repeated cross-sectional data (2008, 2013);<br>- Sample size: 42,706;<br>- Population: individuals, representative at national level and at the level of administrative areas (27 departamentos);<br>- Missing data: unclear;<br>- Source: Estudio Nacional de Consumo de Sustancias Psicoactivas en Colombia.
Methods	Logistic regression
Dependent variables	- Participation (unclear how it was defined).

Price/tax variable(s)	State-level (9 departamentos) prices constructed from household-level unit values (2006–2007) and CPI for tobacco/cigarettes;<br>Deflator: unclear<br>Source: Encuesta Nacional de Ingresos y Gastos; source of CPI data not reported.
Covariates	Household head (0,1); alcohol consumption (0,1); ever trying marijuana, sex, marital status; employment (employed, unemployed, studying); education (< primary, primary, secondary and, ≥ tertiary); SES (low, mid, high); year-month and municipality fixed effects.
Misspecification tests	None reported.
Sensitivity analyses	Models re-estimated excluding respondents who were ≤ 10, 12, 14 and 16 years at interview.
Results	Participation own-price elasticity:<br>- all: -0.66 (95%CI -1.31, -0.01)<br>- Socioeconomic Status<br>- low: -0.70 (95%CI -1.42, 0.01)<br>- mid: -0.62 (95%CI -1.26, 0.01)<br>- high: -0.72 (95%CI -1.41, -0.03)<br>*Summary of findings*: no evidence of any statistically or economically significant socioeconomic differences in price responsiveness for cigarette smoking participation. Initiation and cessation also explored; no differences between socioeconomic categories were found.<br><br>Source: Tables 2, 3.
Limitations	- dependent variable not clearly defined;<br>- unclear adjustment for inflation;<br>- unclear how missing data were dealt with;<br>- no testing for misspecification;<br>- limited variation in space (9 states) and time (2 survey cycles);<br>- no formal assessment of socioeconomic differences in price responsiveness;<br>- imprecision: uncertainty intervals very wide.<br>Risk of bias: very high
Funding, competing interests reported?	Funding: International Development Research Center (IDRC);<br>Competing interests: disclosed (none reported).

Guindon GE, Paraje GR, Chaloupka FJ. Association of Tobacco Control Policies With Youth Smoking Onset in Chile. JAMA Pediatr 2019;173(8):754-62. doi: 10.1001/jamapediatrics.2019.1500

Country/Journal	- Chile<br>- JAMA Pediatr
Data	- Type: retrospective constructed from cross-sectional data (2001, 2003, 2005, 2007, 2009, 2011, 2013, 2015);<br>- Sample size: 181,624 survey respondents in 8751 Chilean secondary schools;<br>- Population: nationally and regionally representative urban school-based survey (grades 8-12); main analyses restricted to 16 to 19 years at interview;<br>- Missing data: listwise deletion;<br>- Source: Encuesta de Población Escolar de Chile.

Methods	Survival/duration analyses:<br>- discrete-time hazard models and a complementary loglog specification;<br>- functional form of baseline hazard function: cubic polynomial specification.
Dependent variables	- age at smoking onset (how old were you when you smoked for the first time?); month/year of onset randomly selected using uniform distribution; sensitivity analyses using mid-point;<br>- age at first risk of starting: 8
Price/tax variable(s)	Cigarette component of the Consumer Price Index (CPI), in real terms;<br>Deflator: CPI all-items<br>Source: Instituto Nacional de Estadísticas
Covariates	Sex; mother's educational level; type of school; 2006 tobacco control law; regions, alcohol prices.
Misspecification tests	None reported.
Sensitivity analyses	- Alternative functional form for the baseline hazard function (dummy specification for time at risk, measured in years);<br>- estimated discrete-time split population models;<br>- estimated all models with and without sampling weights;<br>- included measures of calendar time (1. fourth-order polynomial, measured in years; 2. controlled for differing birth cohorts).
Results	Initiation own-price elasticity, cigarettes:<br>- all: -0.40 (95%CI -0.45, -0.36)<br>- Mother's educational level:<br>- primary or less: -0.41 (95%CI -0.48, -0.34)<br>- secondary or less: -0.44 (95%CI -0.49, -0.38)<br>- more than secondary: -0.36 (95%CI -0.42, -0.30)<br>- Type of school:<br>- public -0.41 (95%CI -0.47, -0.35)<br>- subsidized: -0.44 (95%CI -0.50, -0.38)<br>- private: -0.30 (95%CI -0.39, -0.20)<br>*Summary of findings*: no evidence of any statistically or economically significant socioeconomic differences in price responsiveness for cigarette smoking initiation.<br><br>Source: Table 2, model 5.
Limitations	- no testing for misspecification;<br>- non informative censoring.<br>Risk of bias: moderate
Funding, competing interests reported?	Funding: International Development Research Center (IDRC);<br>Competing interests: disclosed (none reported).

Gonzalez-Rozada M, Ramos-Carbajales A. Implications of raising cigarette excise taxes in Peru. Rev Panam Salud Publica 2016;40(4):250-55.

Country/Journal	- Peru<br>- Pan American Journal of Public Health

Data	- Type: cross-sectional (2008-2009);<br>- Sample size: 3153 hh with positive cigarette expenditures;<br>- Population: hh, representative at national level and rural and urban-level;<br>- Missing data: unclear;<br>- Source: Encuesta Nacional de Presupuestos Familiares.
Methods	Deaton's two-equation system of budget shares and unit values
Dependent variables	- share of the budget devoted to cigarettes.
Price/tax variable(s)	Unit values (expenditures/quantity consumed) at cluster-level (clusters not defined; # of clusters not reported)<br>Deflator: unclear<br>Source: n/a
Covariates	# of persons in hh; % of men and women > 18 years in hh.
Misspecification tests	None reported.
Sensitivity analyses	Estimations were performed using households and individuals as the unit of analysis.
Results	Total own-price elasticity, cigarettes:<br>- all: -0.68 (95%CI -1.09, -0.26)<br>- Household total expenditures<br>- tercile 1, high: -0.81 (95%CI -1.09, -0.53)<br>- tercile 2, mid: -0.57 (95%CI -0.70, -0.44)<br>- tercile 3, low: -0.75 (95%CI -1.03, -0.47)<br>*Summary of findings*: no evidence of any statistically significant socioeconomic differences in price responsiveness for total cigarette consumption; estimates suggest a possible u-shaped association between socioeconomic status and price responsiveness.<br><br>Source: Table 4, bottom panel.
Limitations	- no testing for misspecification;<br>- unclear how missing data/outliers were dealt with;<br>- unclear adjustment for inflation;<br>- clusters not defined; # of clusters not reported; # of household with positive cigarette expenditures within each cluster not clearly reported;<br>- imprecision: uncertainty intervals very wide;<br>- no formal assessment of socioeconomic differences in price responsiveness.<br>Risk of bias: high
Funding, competing interests reported?	Funding: International Development Research Center (IDRC);<br>Competing interests: disclosed (none reported).

Chavez R. [Price elasticity of demand for cigarettes and alcohol in Ecuador, based on household data]. Rev Panam Salud Publica 2016;40(4):222-28.

Country/Journal	- Ecuador<br>- Rev Panam Salud Publica

Data	- Type: cross-sectional (Apr 2011 - Mar 2012);<br>- Sample size: 39,617 hh.<br>- Population: hh, representative at national level at rural and urban-level;<br>- Missing data: not reported;<br>- Source: Encuesta Encuesta Nacional de Ingresos y Gastos de Hogares Urbanos y Rurales (ENIGHUR).
Methods	Deaton's two-equation system of budget shares and unit values
Dependent variables	- share of the budget devoted to cigarettes;<br>- share of the budget devoted to alcohol (not clearly defined).
Price/tax variable(s)	Unit values (expenditures/quantity consumed) at cluster-level (624 *parroquias* [parishes])<br>Deflator: unclear<br>Source: n/a
Covariates	Not clearly presented.
Misspecification tests	None reported.
Sensitivity analyses	None reported.
Results	Total own-price elasticity, cigarettes:<br>- all: -0.87 (95%CI -0.60, -0.21)<br>- Household total expenditures<br>- tercile 1 (low): -0.25 (95%CI -58.7, 58.2)<br>- tercile 2: -1.14 (95%CI -2.10, -0.18)<br>- tercile 3: -1.25 (95%CI -1.84, -0.66)<br>*Summary of findings*: no evidence of any statistically or economically significant socioeconomic differences in price responsiveness for total cigarette consumption.<br><br>Source: Tables 3 and 4.
Limitations	- no testing for misspecification;<br>- no sensitivity analyses;<br>- unclear how missing data/outliers were dealt with;<br>- covariates not clearly presented;<br>- # of household with positive cigarette/alcohol expenditures within each cluster not clearly reported;<br>- # of clusters with at least one household with positive cigarette/alcohol expenditures not clearly reported;<br>- unclear adjustment for inflation;<br>- imprecision: uncertainty intervals very wide;<br>- no formal assessment of socioeconomic differences in price responsiveness.<br>Risk of bias: high
Funding, competing interests reported?	Funding: Escuela de Negocios de la Universidad Adolfo Ibáñez, American Cancer Society; International Development Research Center (IDRC);<br>Competing interests: disclosed (none reported).

Franco-Churruarin F, Gonzalez-Rozada M. The impact of cigarette price increases on the prevalence of daily smoking and initiation in Mexico. A Tobacconomics Research Report. Chicago, IL: Tobacconomics, Health Policy Center, Institute for Health Research and Policy, University of Illinois Chicago, 2021.

Country/Journal	- Mexico<br>- Report (University of Illinois Chicago)
Data	- Type: Cross-sectional (2015)<br>- Sample size: 14,664 individuals;<br>- Population: individuals ≥ 15 years, representative at national level;<br>- Missing data: not reported;<br>- Source: Global Adult Tobacco Survey (GATS)
Methods	Two-part model:<br>- Participation: probit (prices in ln)
Dependent variables	- Participation: daily smokers (individuals who self-reported smoking and who smoked a positive number of cigarettes each day)
Price/tax variable(s)	1) Self-reported price paid for the last purchase assigned to smokers; random regression imputation (stochastic regression imputation) to assign price to non-smokers;<br>2) Mean self-reported price by primary sampling unit (PSU) assigned to smokers and non-smokers (no. of PSUs not reported).<br>Deflator: n/a<br>Source: n/a
Covariates	Wealth index; sex; age; rural/urban; student; employment (employed, unemployed, out of labour force).
Misspecification tests	Rivers-Vuong test statistic
Sensitivity analyses	Used two measures of price constructed from self-reported prices.
Results	Participation own-price elasticity, cigarettes*:<br>– Wealth quartiles:<br>- Q1 (low): -0.44 (95%CI -0.60, -0.27)<br>- Q2: -0.41 (95%CI -0.56, -0.26)<br>- Q3: -0.39 (95%CI -0.54, -0.25)<br>- Q4: -0.37 (95%CI -0.51, -0.24)<br><br>- Q1 (low): -0.45 (95% CI -0.60, -0.29)<br>- Q2: -0.42 (95% CI 0.38, -1.22)<br>- Q3: -0.41 (95% CI -0.55, -0.27)<br>- Q4: -0.39 (095% CI -0.52, -0.27)<br><br>* The first set of estimates were obtained using a measure of prices based on self-reported price paid for the last purchase assigned to smokers; and random regression imputation (stochastic regression imputation) to assign price to non-smokers; the second set of estimated were obtained using mean self-reported price by primary sampling unit assigned to smokers and non-smokers.<br><br>*Summary of findings*: no evidence of any statistically or economically significant socioeconomic differences in price responsiveness for cigarette participation.<br><br>Source: Tables 6, B3

Limitations	- unclear how missing data were handled;<br>- no. of clusters not reported;<br>- imprecision: uncertainty intervals fairly wide;<br>- no formal assessment of socioeconomic differences in price responsiveness;<br>- conclusion not supported by results.<br>Risk of bias: high
Funding, competing interests reported?	Funding: Bloomberg Philanthropies<br>Competing interests: not disclosed.

Sáenz de Miera Juárez B, Guerrero López CM, Zúñiga Ramiro J, et al. Impuestos al tabaco y políticas para el control del tabaco en Brasil, México y Uruguay - resultados para México. México: Fundación InterAmericana del Corazón México (FIC México) 2013.

Country/Journal	- Mexico<br>- Report (Fundación InterAmericana del Corazón México)
Data	- Type: repeated cross-sectional (1994, 1996, 1998, 2000, 2002, 2004, 2005, 2006, 2008, 2010, 2012)<br>- Sample size: 196,089 hh (12,815 hh in 1994, 13,096 in 1996, 10,134 in 1998, 10,108 in 2000, 17,167 in 2002, 22,595 in 2004, 23,174 in 2005, 20,875 in 2006, 29,468 in 2008, 27,655 in 2010, 9,002 in 2012).<br>- Population: hh, representative at national level;<br>- Missing data: not reported;<br>- Source: Encuesta Nacional de Ingresos y Gastos de los Hogares (ENIGH)
Methods	Two-part model:<br>- Participation: probit (prices in ln)<br>- Consumption: weighted-OLS (functional form: ln-ln)
Dependent variables	- Participation: positive cigarette expenditures;<br>- Consumption: monthly cigarette consumption (calculated from weekly cigarette consumption measured in kg using conversion factor 1 kg = 800 cigarettes)
Price/tax variable(s)	Predicted unit values (expenditures/quantity consumed) using categorical indicators of household income (quintiles), rural/urban status; state;<br>Deflator: unclear<br>Source: n/a
Covariates	Income (total hh income per person, ln); hh head's education, sex, age, age squared; alcohol use; number and squared number of adults in the hh; wave dummies.
Misspecification tests	None reported.
Sensitivity analyses	None reported.

Results	Total own-price elasticity, cigarettes:<br>- all: -0.57 (95%CI -0.47, -0.60)<br>- tercile 1: -0.60 (95%CI -0.41, -0.79)<br>- tercile 2: -0.60 (95%CI -0.42, -0.77)<br>- tercile 3: -0.55 (95%CI -0.37, -0.73)<br>Participation own-price elasticity, cigarettes*:<br>- all: -0.17 (p-value < 0.01)<br>- tercile 1: -0.20 (p < 0.01)<br>- tercile 2: -0.20 (p < 0.01)<br>- tercile 3: -0.11 (p < 0.05)<br>Consumption own-price elasticity, cigarettes*:<br>- all: -0.40 (p < 0.01)<br>- tercile 1: -0.40 (p < 0.01)<br>- tercile 2: -0.39 (p < 0.01)<br>- tercile 3: -0.44 (p < 0.01)<br>* measures of uncertainty not reported<br>*Summary of findings*: no evidence of any statistically or economically significant socioeconomic differences in price responsiveness for total cigarette consumption.<br><br>Source: Tables 4.3, 4.4.
Limitations	- no testing for misspecification;<br>- no sensitivity analyses;<br>- no account for measurement error or endogeneity;<br>- unclear adjustment for inflation;<br>- imprecision: uncertainty intervals fairly wide; not all uncertainty intervals reported;<br>- no formal assessment of socioeconomic differences in price responsiveness.<br>Risk of bias: high
Funding, competing interests reported?	Funding: International Development Research Center (IDRC)<br>Competing interests: not disclosed.

Saenz-de-Miera B, Thrasher JF, Chaloupka FJ, et al. Self-reported price of cigarettes, consumption and compensatory behaviours in a cohort of Mexican smokers before and after a cigarette tax increase. Tob Control 2010;19(6):481-7. doi: 10.1136/tc.2009.032177

Country/Journal	- Mexico<br>- Tobacco Control
Data	- Type: longitudinal survey (wave 1: Sep-Nov 2006, wave 2: Nov-Dec 2007);<br>- Sample size: wave 1, 1079; wave 2756;<br>- Population: current smokers (≥ 18 years) randomly selected from four large Mexican cities (Mexico City, Guadalajara, Tijuana and Ciudad Juarez);<br>- Missing data: unclear;<br>- Source: International Tobacco Control Policy Evaluation Mexico Survey (ITC-Mexico).
Methods	t-tests (average number of cigarettes smoked per day at baseline and follow-up)<br>Logistic regressions (quitting at follow-up)
Dependent variables	- Average number of cigarettes smoked per day at baseline and follow-up;<br>- Quitting at follow-up.

Price/tax variable(s)	Pre-post tax changes: tax increased from 110% of the price to the retailer to 140%; magnitude of price change is unclear as authors incorrectly assumed that "increase in taxes is expected to increase final prices at least in the same proportion." Average self-reported cigarette pack price increased among smokers whose last purchase was a pack at both survey waves was 12.7% (inflation adjusted).<br>Deflator: unclear<br>Source: unclear
Covariates	Average number of cigarettes smoked per day at baseline and follow-up: unclear, likely not adjusted;<br>Quitting at follow-up: age, sex, marital status, education, employment status, income, smoking intensity, quit attempts in past year; plan to quit in next six months.
Misspecification tests	None reported.
Sensitivity analyses	Additional analyses of cigarette consumption changes using bivariate and multi-variable random effects linear regression models, adjusted for intraindividual correlation of data from participants observed at both waves;<br>Using the entire baseline and follow-up samples, consumption was regressed on time (ie, baseline vs follow-up) and other study variables. Results indicated that time was inversely associated with consumption, and planning to quit was the only other statistically significant predictor of consumption in multivariable models.
Results	Average number of cigarettes smoked per day at baseline and follow-up (percentage change*)<br>All: -30%<br>- Education level<br>- primary graduate or less: -29%<br>- secondary graduate: -27%<br>- high school graduate or more: -33%<br>- Monthly household income:<br>- low: -27%<br>- mid: -35%<br>- high: -27%<br>Quitting at follow-up (relative risks):<br>- Education level (ref: primary graduate or less)<br>- secondary graduate: 1.3 (95%CI 0.5, 3.2)<br>- high school graduate or more: 1.5 (95%CI 0.6, 3.8)<br>- Monthly household income (ref: low)<br>- mid: 1.03 (95%CI 0.48, 2.2)<br>- high: 0.55 (95%CI 0.2, 1.5)<br>* measures of uncertainty not reported;<br>*Summary of findings*: no evidence of any statistically or economically significant socioeconomic differences in price responsiveness for cigarette consumption or cessation.<br><br>Source: Tables 3 and 4.

Limitations	- no testing for misspecification;<br>- unclear how missing data were dealt with;<br>- non-random attrition;<br>- unclear how change in taxes affected price;<br>- unclear adjustment for inflation;<br>- imprecision: uncertainty intervals very wide; not all uncertainty intervals reported;<br>- no formal assessment of socioeconomic differences in price responsiveness;<br>- limited generalizability of findings (respondents selected for 4 urban centres).<br>Risk of bias: high
Funding, competing interests reported?	Funding: Consejo Nacional de Ciencia y Tecnología Mexico; US National Institute of Health;<br>Competing interests: disclosed (none reported).

– Asia: Eastern Asia

Huang J, Zheng R, Chaloupka FJ, et al. Differential responsiveness to cigarette price by education and income among adult urban Chinese smokers: findings from the ITC China Survey. Tob Control 2015;24 Suppl 3:iii76-iii82. doi: 10.1136/tobaccocontrol-2014-052091

Country/Journal	- China<br>- Tobacco Control
Data	- Type: repeated cross-sectional (2009; 2015)<br>- Sample size: 9545 (2009); 11,489 (2015)<br>- Population: individual, 15+ years old; representative at national level;<br>- Missing data: listwise deletion; unclear how many observations were missing and how outliers were health with;<br>- Source: Global Adult Tobacco Survey
Methods	Multivariable linear analyses using generalized estimating equations (GEE)
Dependent variables	- average number of cigarettes consumed per day (in ln)
Price/tax variable(s)	Self-reported price-per-pack, aggregated at cluster level (city district level); # not reported<br>Deflator: prices adjusted for inflation but deflator not reported<br>Source: not reported
Covariates	Sex; education; employment; income; age; marital status; interview years; city
Misspecification tests	None reported
Sensitivity analyses	None reported
Results	Consumption own-price elasticity, cigarettes:<br>– All: -0.12 (95%CI -0.16, -0.09)<br>– Income<br>- high: -0.15 (95%CI -0.21, -0.09)<br>- mid: -0.14 (95%CI -0.20, -0.09)<br>- low: -0.11 (95%CI -0.22, 0.00)<br>– Education:<br>- Post-secondary: -0.11 (95%CI -0.18, -0.05)<br>- High school: -0.11 (95%CI -0.16, -0.06)<br>- Less than high school: -0.14 (95%CI -0.26, -0.03)<br>*Summary of findings*: no evidence of any statistically or economically significant socioeconomic differences in price responsiveness for cigarette smoking.<br>Source: Tables 3 and 4.
Limittations	- no testing for misspecification;<br>- no sensitivity analyses;<br>- unclear reporting of missing data/outliers;<br>- # of clusters not reported.<br>Risk of bias: high
Funding, competing interests reported?	Funding: US National Cancer Institute, Roswell Park Transdisciplinary Tobacco Use Research Centre, Robert Wood Johnson Foundation, Canadian Institutes for Health Research, Chinese Centre for Disease Control and Prevention;<br>Competing interests: disclosed (none reported)

Chen Y, Xing W. Quantity, quality, and regional price variation of cigarettes: Demand analysis based on a household survey in China. China Economic Review 2011;22(2):221-32. doi: 10.1016/j.chieco.2011.01.004

Country/Journal	- China<br>- China Economic Review
Data	- Type: repeated cross-sectional (1999-2001)<br>- Sample size: 11,889 hh (out of 16,441)<br>- Population: urban hh, representativeness unclear;<br>- Missing data: unclear<br>- Source: Urban Household Income and Expenditure Survey
Methods	Deaton's two-equation system of budget shares and unit values
Dependent variables	- Share of the budget devoted to cigarettes
Price/tax variable(s)	Unit values (expenditures/quantity consumed) at cluster-level (24 clusters; 8 provinces, 3 years)<br>Deflator: unclear<br>Source: n/a
Covariates	Not clearly described.
Misspecification tests	Unclear
Sensitivity analyses	None relevant to socioeconomic differences in price responsiveness.
Results	Total own-price elasticity, cigarettes*:<br>Year: 1999-2001<br>– all: -0.43<br>Household income:<br>- tercile 1 (low): -0.46<br>- tercile 2: -0.42<br>- tercile 3: -0.42<br>Year: 1999; 2000; 2001<br>– all: -0.48; -0.56; -0.35<br>Household income:<br>- tercile 1 (low): -0.70; -0.70; -0.37<br>- tercile 2: -0.57; -0.61; -0.38<br>- tercile 3: -0.43; -0.51; -0.32<br>* measures of uncertainty/significance level not reported.<br>*Summary of findings*: no evidence of any statistically or economically significant socioeconomic differences in price responsiveness for cigarette smoking.<br><br>Source: Table 6
Limittations	- unclear reporting: covariates;<br>- no testing for misspecification;<br>- no sensitivity analyses;<br>- unclear how missing data/outliers were dealt with;<br>- very low # of clusters;<br>- measures of uncertainty/significance level not reported;<br>- no formal assessment of socioeconomic differences in price responsiveness.<br>Risk of bias: very high
Funding, competing interests reported?	Funding: World Health Organization (WHO), Bloomberg Foundation and Johns Hopkins University;<br>Competing interests: not reported

– **Asia: South-eastern Asia**

Cheng KJG, Estrada MAG. Price Elasticity of cigarette smoking demand in the Philippines after the 2012 Sin Tax Reform Act. Prev Med 2020;134:106042. doi: 10.1016/j.ypmed.2020.106042

Country/Journal	- Philippines<br>- Preventive Medicine
Data	- Type: repeated cross-sectional (2009; 2015)<br>- Sample size: 9,545 (2009); 11,489 (2015)<br>- Population: individual, 15+ years old; representative at national level; , sampled using a multistage geographically clustered sampling design;<br>- Missing data: listwise deletion; unclear how many observations were dropped;<br>- Source: Global Adult Tobacco Survey.
Methods	Two-part model:<br>- Participation: probit (functional form unclear); IV-probit<br>- Consumption: OLS; two-stage least squares (functional form unclear)
Dependent variables	- Participation: unclear ('smoked daily and less than daily')<br>- Consumption: 'number of sticks smoked daily'
Price/tax variable(s)	Average price-per-stick (quantity in pack/price of recent purchase) of each respondent's primary sampling unit or type of residence (if primary sampling unit not available)<br>- Instrumental variable: weighted tax-per-stick computed using the volume of removals reported by Philippine Bureau of Internal Revenue (price observations that were equivalent or higher than the computed weighted tax-per-stick were dropped)<br>Deflator: unclear;<br>Source: Philippine Bureau of Internal Revenue (BIR).
Covariates	Sex; education; employment status; wealth (constructed using principal components analysis); age; area of residence (urban/rural); exposure to media relating to the dangers of smoking cigarettes, addiction.
Misspecification tests	Some endogeneity tests were conducted.
Sensitivity analyses	Results sensitive to adjusting for survey cycle; results not reported.

Results	Participation own-price elasticity, cigarettes:<br>– Education:<br>- < elementary: -0.11 (P < 0.01)<br>- elementary: -0.08 (P < 0.01)<br>- > elementary, ≤ high school: -0.87 (P < 0.01)<br>- > high school: -1.36 (P < 0.01)<br>– Wealth:<br>- tertile 1 (low): -1.79 (P < 0.01)<br>- tertile 2: -1.40 (P < 0.01)<br>- tertile 3: -0.84 (P < 0.01)<br>Consumption own-price elasticity, cigarettes:<br>– Education:<br>- < elementary: -1.0 (P < 0.1)<br>- elementary: not reported (P > 0.1)<br>- > elementary, ≤ high school: 0.19 (P < 0.05)<br>- > high school: not reported (P > 0.1)<br>– Wealth:<br>- tertile 1 (low): 0.14 (P < 0.05)<br>- tertile 2: 0.16 (P < 0.1)<br>- tertile 3: not reported (P > 0.1)<br><br>*Summary of findings*: it is unclear if there were any statistically or economically significant socioeconomic differences in price responsiveness.<br><br>Source: Table 5
Limittations	- unclear reporting of missing data/outliers;<br>- # of clusters not reported;<br>- no testing for misspecification;<br>- measures of uncertainty not reported;<br>- results extremely sensitive to alternative specifications;<br>- non-sensical conditional own-price elasticities;<br>- no formal assessment of socioeconomic differences in price responsiveness.<br>Risk of bias: very high
Funding, competing interests reported?	Funding: US National Cancer Institute, Roswell Park Transdisciplinary Tobacco Use Research Centre, Robert Wood Johnson Foundation, Canadian Institutes for Health Research, Chinese Centre for Disease Control and Prevention;<br>Competing interests: disclosed (none reported)

Quimbo SLA, Casorla AA, Miguel-Baquilod M, Medalla, FM, Chaloupka, FJ. The Economics of Tobacco and Tobacco Taxation in the Philippines. Paris: International Union Against Tuberculosis and Lung Disease, 2012.

Country/Journal	- Phillipines<br>- Report, International Union Against Tuberculosis and Lung Disease
Data	- Type: cross-sectional (2003);<br>- Sample size: ≈ 42,000 hh (21,942 tobacco spending hh used in analyses)<br>- Population: hh, representative at national level;<br>- Missing data: not reported;<br>- Source: Family Income and Expenditure Survey (FIES)
Methods	Two-Stage Least Squares (instrument: regions [no description or no. of regions provided])
Dependent variables	Mean household cigarette consumption, per household member (consumption obtained from cigarette expenditures / average price at province-level)

Price/tax variable(s)	Average cigarette prices in 2003 at province-level (no. of provinces not clearly reported; likely 17 or 56 provinces); data source: National Statistics Office<br>Deflator: n/a<br>Source: n/a
Covariates	Total household expenditures; household head's age, sex, employment status, and education; expenditures on insurance (proxy for risk taking).
Misspecification tests	Hausman Test of Endogeneity; Chow Tests
Sensitivity analyses	Re-estimate all models using reported cigarette expenditures as dependent variable an dextracted the price of elasticity of demand (indirect method). Results were similar to those produced by the direct method.
Results	Consumption own-price elasticity, cigarettes*<br>Household expenditures:<br>- All: -0.87 (P < 0.01)<br>- deciles 1-3 (low): -1.09 (P < 0.01)<br>- deciles 4-6: -0.80 (P < 0.01)<br>- deciles 7-9: -0.74 (P < 0.01)<br>- decile10: -0.52 (P < 0.01)<br>* measures of uncertainty not reported.<br>*Summary of findings*: Point estimates suggest that lower-SES households were more responsive to price; differences were large enough to be economically meaningful.<br><br>Source: Tables 6.1, A.8
Limitations	- unclear how missing data/outliers were handled;<br>- no. of clusters (provinces) not clearly reported;<br>- no account for quality;<br>- limited variation in space (17 or 57 provinces) and time (no variation in time);<br>- unclear if instrument is valid; regions likely correlated with dependent variable;<br>- imprecision: uncertainty intervals not reported;<br>- no formal assessment of socioeconomic differences in price responsiveness.<br>Risk of bias: very high
Funding, competing interests reported?	Funding: Bloomberg Philanthropies; Bill and Melinda Gates Foundation<br>Competing interests: not disclosed.

Jankhotkaew J, Pitayarangsarit S, Chaiyasong S, et al. Price elasticity of demand for manufactured cigarettes and roll-your-own cigarettes across socioeconomic status groups in Thailand. Tob Control 2021;30(5):542-47. doi: 10.1136/tobaccocontrol-2019-055480

Country/Journal	- Thailand<br>- Tobacco Control
Data	- Type: cross-sectional (2017)<br>- Sample size: 98 685 (80,965 after listwise deletion);<br>- Population: individuals ≥ 15 years, representative at national level;<br>- Missing data: listwise deletion (18%);<br>- Source: The smoking and drinking behaviour survey, National Statistical Office.
Methods	Two-part model:<br>- Participation: probit (prices in ln)<br>- Consumption: OLS

Dependent variables	- Participation: no clear definition provided;<br>- Consumption: “number of cigarette sticks bought in the last purchase”
Price/tax variable(s)	Cigarettes/roll-your-own: unit values at cluster level (village); no.of clusters not reported.<br>Deflator: n/a<br>Source: n/a
Covariates	Income; sex; age, highest educational achievement; marital status; employment status; rural/urban; region.
Misspecification tests	None
Sensitivity analyses	Estimated three specifications with different cigarette type.
Results	Participation own-price elasticity, cigarettes*:<br>– Income tertiles:<br>- Low: 0.08 ($P < 0.05$)<br>- Mid: 0.06 ($P < 0.05$)<br>- High: 0.02 ($P < 0.05$)<br>Consumption own-price elasticity, cigarettes*:<br>– Income tertiles:<br>- Low: -0.61 ($P < 0.05$)<br>- Mid: -0.57 ($P < 0.05$)<br>- High: -0.49 ($P < 0.05$)<br>Total own-price elasticity, cigarettes*:<br>– Income tertiles:<br>- Low: -0.53 ($P < 0.05$)<br>- Mid: -0.51 ($P < 0.05$)<br>- High: -0.47 ($P < 0.05$)<br>Participation own-price elasticity, roll-your-own*:<br>– Income tertiles:<br>- Low: -0.15 ($P < 0.05$)<br>- Mid: -0.12 ($P < 0.05$)<br>- High: -0.19 ($P < 0.05$)<br>Consumption own-price elasticity, roll-your-own*:<br>– Income tertiles:<br>- Low: -0.25 ($P < 0.05$)<br>- Mid: -0.19 ($P < 0.05$)<br>- High: -0.18 ($P < 0.05$)<br>Total own-price elasticity, roll-your-own*:<br>– Income tertiles:<br>- Low: -0.40 ($P < 0.05$)<br>- Mid: -0.36 ($P < 0.05$)<br>- High: -0.27 ($P < 0.05$)<br>* measures of uncertainty not reported.<br>*Summary of findings*: No evidence of any statistically or economically significant socioeconomic differences in price responsiveness for cigarette participation or consumption. Point estimates suggest that lower socioeconomic status individuals were more responsive to price for roll-your-own participation; it is unclear if differences were large enough to be statistically or economically significant.<br><br>Source: Tables 6, S1

Limitations	- unclear description of dependent variables;<br>- 18% of eligible respondent excluded because of missing data;<br>- no testing for misspecification;<br>- clusters not clearly defined; no. of clusters not reported; no. of individual with positive cigarette/roll-your-own expenditures within each cluster not reported; no. of clusters with at least one individual with positive cigarette/roll-your-own expenditures not reported;<br>- no account for measurement error or quality;<br>- nonsensical positive conditional own-price elasticities;<br>- imprecision: measures of uncertainty not reported;<br>- no formal assessment of socioeconomic differences in price responsiveness.<br>Risk of bias: very high
Funding, competing interests reported?	Funding: disclosed (none reported)<br>Competing interests: disclosed (none reported)

— Asia: Southern Asia

Huque R, Abdullah SM, Hossain N. Cigarette Demand Analysis: Bangladesh. Estimating Elasticity of Cigarette Smoking in Bangladesh Using GATS Data. Dhaka, Bangladesh: ARK Foundation, 2021.

Country/Journal	- Bangladesh<br>- Report, ARK Foundation
Data	- Type: cross-sectional (2009, 2017)<br>- Sample size: 9,629 individuals (2009); 12,783 individuals (2017)<br>- Population: individuals ≥ 15 years, representative at national level;<br>- Missing data: not reported;<br>- Source: Global Adult Tobacco Survey (GATS)
Methods	Two-part model:<br>- Participation: probit (prices in ln)<br>- Consumption: unclear
Dependent variables	- Participation: no clear definition provided; "people who smoke cigarettes daily or less than daily"<br>- Consumption: no clear definition provided; "number of sticks smoked"
Price/tax variable(s)	Unit values at cluster level (weighted by individual cigarette expenditure); clusters not defined, no.of clusters not reported.<br>Deflator: n/a<br>Source: n/a
Covariates	Biri unit values at cluster-level; wealth index; sex; age; rural/urban; education; employment type; age; "smoking restrictions in homes (self-imposed) and workplaces (legislation or regulation imposed by authority), exposure to smoking warnings, advertising, promotional activities, and perceptions;" unclear how smoking restrictions, exposure to warnings/advertising were operationalized.
Misspecification tests	None
Sensitivity analyses	Estimated three specifications with different covariates.
Results	Participation own-price elasticity, cigarettes:<br>– Wealth quintiles:<br>- Q1-Q3 (low): -0.86 (95%CI -1.19, -0.53)<br>- Q4-Q5: -0.35 (95%CI -0.60, -0.10)<br>Consumption own-price elasticity, cigarettes:<br>– Wealth quintiles:<br>- Q1-Q3 (low): -0.04 (95%CI -0.14, 0.06)<br>- Q4-Q5: -0.04 (95%CI -0.10, 0.02)<br><br>*Summary of findings*: Point estimates suggest that lower socioeconomic status individuals were more responsive to price for cigarette participation; differences were large enough to be economically significant. No formal assessment of socioeconomic differences in price responsiveness. Uncertainty intervals suggest that differences were not statistically significant. No evidence of any statistically or economically significant socioeconomic differences in price responsiveness for cigarette consumption.<br><br>Source: Table 6, model 3

Limitations	- unclear description of dependent variables;<br>- covariates not clearly described;<br>- no testing for misspecification;<br>- clusters not clearly defined; no. of clusters not reported;<br>- no account for measurement error or quality;<br>- imprecision: uncertainty intervals fairly wide;<br>- no formal assessment of socioeconomic differences in price responsiveness.<br>Risk of bias: very high
Funding, competing interests reported?	Funding: Bloomberg Philanthropies; University of Illinois Chicago<br>Competing interests: disclosed (none reported)

Del Carmen G, Fuchs A, Genoni ME. The Distributional Impacts of Cigarette Taxation in Bangladesh. Policy Research Working Paper No. 8580. Washington, DC: World Bank, 2018.

Country/Journal	- Bangladesh<br>- World Bank Working Paper
Data	- Type: cross-sectional (2016-2017)<br>- Sample size: 46,000 hh (approx.)<br>- Population: hh, representative at national level;<br>- Missing data: not reported;<br>- Source: Household Income and Expenditure Survey
Methods	Quadratic Almost Ideal Demand System, QUAIDS
Dependent variables	- share of the budget devoted to cigarettes;<br>- share of the budget devoted to bidis;<br>- share of the budget devoted to betel leaf;<br>- share of the budget devoted to betel nut;<br>- share of the budget devoted to rolled betel leaf.
Price/tax variable(s)	Median unit values (monthly expenditure/quantity purchased) at district level in urban and rural areas. In the cases where the number of observations was less than 30, the medians of the full districts were used. Number of districts not reported.<br>Deflator: none<br>Source: n/a
Covariates	hh size; number of males (15+ years) in hh; age, sex, religion, educational attainment of hh head; urban/rural
Misspecification tests	Unclear
Sensitivity analyses	Unclear

Results	Total own-price elasticity, cigarettes*:<br>- Decile 1 (poorest): -1.36<br>- Decile 2: -1.33<br>- Decile 3: -1.33<br>- Decile 4: -1.29<br>- Decile 5: -1.33<br>- Decile 6: -1.27<br>- Decile 7: -1.24<br>- Decile 8: -1.25<br>- Decile 9: -1.25<br>- Decile 10: -1.23<br>Total own-price elasticity, bidis*:<br>- Decile 1 (poorest): -1.14<br>- Decile 2: -1.26<br>- Decile 3: -1.18<br>- Decile 4: -1.26<br>- Decile 5: -1.19<br>- Decile 6: -1.18<br>- Decile 7: -1.21<br>- Decile 8: -1.18<br>- Decile 9: -1.27<br>- Decile 10: -1.29<br>* measures of uncertainty/significance level not reported<br>*Summary of findings*: no evidence of any statistically or economically significant socioeconomic differences in price responsiveness for cigarette or bidi smoking.<br><br>Source: Table 3
Limittations	- no testing for misspecification;<br>- no sensitivity analyses;<br>- unclear how missing data/outliers were dealt with;<br>- no account for measurement error or endogeneity;<br>- # of household with positive cigarette/bidi expenditures within each cluster not clearly reported; # of clusters with at least one household with positive cigarette/bidi expenditures not clearly reported;<br>- imprecision: uncertainty intervals not reported;<br>- no formal assessment of socioeconomic differences in price responsiveness.<br>Risk of bias: very high
Funding, competing interests reported?	Funding: World Bank; Bill and Melinda Gates Foundation; Bloomberg Philanthropies.<br>Competing interests: not disclosed.

Nargis N, Ruthbah UH, Hussain AKMG, et al. The price sensitivity of cigarette consumption in Bangladesh: evidence from the International Tobacco Control (ITC) Bangladesh Wave 1 (2009) and Wave 2 (2010) Surveys. Tob Control 2014;23 Suppl 1:i39-47. doi: 10.1136/tobaccocontrol-2012-050835

Country/Journal	- Bangladesh<br>- Tobacco control

Data	- Type: repeated cross-sectional (2009, 2010);<br>- Sample size: 3,652 cigarette smokers, 8,507 total respondents; bidi users excluded from analysis;<br>- Population: individual, 15+ years (smokers and non-smokers), representative at national level;<br>- Missing data: unclear (8% attrition at wave 2);<br>- Source: International Tobacco Control (ITC) Bangladesh Survey.
Methods	Two-part model:<br>- Participation: probit (prices in ln); IV-probit<br>- Consumption: weighted-OLS (functional form: ln-lin); two-stage least squares<br><br>Instrument: tax
Dependent variables	- Participation (unclear how it was defined);<br>- Consumption, average number of cigarettes smoked per day.
Price/tax variable(s)	Prices self-reported by smokers, averaged at "geographic area of residence (village);" # of areas not reported;<br>Deflator: unclear;<br>Source: n/a
Covariates	Household income; household size; sex; age; marital status; household size; education; occupation; household restriction on indoor smoking; survey wave; urban/rural.
Misspecification tests	Ramsey Regression Equation Specification Error Test (RESET)
Sensitivity analyses	Fixed effects models; price effects no longer statistically significant.
Results	Participation own-price elasticity, cigarettes*:<br>– Probit:<br>- all: 0.04 ($P \geq 0.05$)<br>- low SES : 0.01 ($P \geq 0.05$)<br>- mid SES: 0.00 ($P \geq 0.05$)<br>- high SES: 0.13 ($P \geq 0.05$)<br>– IV Probit:<br>- all: -0.29 ($P < 0.001$)<br>- low SES : -0.50 ($P < 0.01$)<br>- mid SES: -0.31 ($P < 0.01$)<br>- high SES: -0.15 ($P \geq 0.05$)<br>Consumption own-price elasticity, cigarettes*:<br>– OLS:<br>- all: -0.21 ($P < 0.01$)<br>- low SES : -0.43 ($P < 0.01$)<br>- mid SES: -0.07 ($P \geq 0.05$)<br>- high SES: -0.14 ($P \geq 0.05$)<br>– 2SLS:<br>- all: -0.20 ($P < 0.01$)<br>- low SES : -0.25 ($P < 0.001$)<br>- mid SES: -0.09 ($P \geq 0.05$)<br>- high SES: -0.21 ($P < 0.01$)<br>* measures of uncertainty not reported<br>*Summary of findings*: it is unclear if there were any statistically or economically significant socioeconomic differences in price responsiveness.<br><br>Source: Table 7

Limitations	- dependent variable not clearly defined;<br>- unclear how missing data/outliers were dealt with;<br>- unclear adjustment for inflation;<br>- clusters not clearly defined; # of clusters not reported; # of self-reported prices per cluster not reported;<br>- imprecision: uncertainty intervals not clearly reported;<br>- socioeconomic differences in price responsiveness not formally assessed;<br>- conclusion not supported by results.<br>Risk of bias: very high
Funding, competing interests reported?	Funding: International Development Research Center (IDRC); Canadian Institutes for Health Research (CIHR); US National Cancer Institute; Roswell Park Transdisciplinary Tobacco Use Research Center; Robert Wood Johnson Foundation; Ontario Institute for Cancer Research<br>Competing interests: disclosed (none reported).

Dauchy EP, John RM. The Effect of Price and Tax Policies on the Decision to Smoke or Use Smokeless Tobacco in India. Prev Sci 2022 doi: 10.1007/s11121-022-01360-w

Country/Journal	- India<br>- Prevention Science
Data	- Type: retrospective constructed from cross-sectional data (2016–17);<br>- Sample size: 74,037 individuals (16,627 ever-smokers)<br>- Population: representative at national level;<br>- Missing data: unclear;<br>- Source: Global Adult Tobacco Survey (GATS)
Methods	Pooled linear probability model with propensity score matching (initiation only):<br>- Baseline: pooled OLS (prices in ln);<br>- First step: probit (full-sample); estimated likelihood of participation based on time-invariant characteristics;<br>- Second step: reweighed data with variance-covariance matrix obtained from first step; estimated impact of price on initiation on subsample narrowed to ever-smokers and their nearest matches;<br>- Studied likelihood of initiation and cessation for individual (i) in period (t) as a function of ln(tobacco prices);<br>Price elasticities based on delta method; used seemingly unrelated regressions (SUR) to simultaneously estimate the model across different subgroups (wealth, age).<br><br>Functional form of duration/time dependency not clearly described; age, age squared included as covariates.
Dependent variables	- smoking initiation; unclear how it was operationalized from “how old were you when you first started smoking tobacco daily?”, and “how many years ago did you first start smoking tobacco daily?”<br>- age at first risk of starting: unclear<br>- cessation; how long has it been since you stopped smoking?
Price/tax variable(s)	Annual average wholesale price indices of cigarettes/bidis (weighted average), and smokeless tobacco from 1980.<br>Deflator: wholesale price indices all-goods<br>Source: Ministry of Commerce and Industry, Government of India.
Covariates	Age; sex; education level; caste; religious group; marital status, wealth.

Misspecification tests	None reported.
Sensitivity analyses	- Estimated additional sets of regressions aimed at controlling for macroeconomic variables that were most likely to affect wholesale prices.
Results	Initiation own-price elasticity, cigarettes/bidis:<br>– all: -0.024 (95%CI -0.024, -0.023)<br>Wealth:<br>- tertile 1 (low): -0.025 (95%CI -0.026, -0.025)<br>- tercile 2: -0.025 (95%CI -0.026, -0.025)<br>- tercile 3: -0.018 (95%CI -0.019, -0.017)<br>Chi-squared test of statistical significance between subgroups: low vs middle, P = 0.37; middle vs high, P < 0.01; low vs high, P < 0.01.<br>Initiation own-price elasticity, smokeless tobacco:<br>– all: -0.024 (95%CI -0.024, -0.023)<br>Wealth:<br>- tertile 1 (low): -0.0004 (95%CI 0.000, 0.000)<br>- tercile 2: -0.0005 (95%CI 0.000, 0.000)<br>- tercile 3: -0.0004 (95%CI 0.000, 0.000)<br>Chi-squared tests of statistical significance between subgroups: P < 0.01 for all comparisons.<br>Cessation own-price elasticity, cigarettes/bidis:<br>– all: 0.022 (95% CI 0.017, 0.026)<br>Wealth:<br>- tertile 1 (low): 0.022 (95%CI 0.014, 0.029)<br>- tercile 2: 0.023 (95%CI 0.016, 0.030)<br>- tercile 3: 0.0211 (95% CI 0.012, 0.030)<br>Chi-squared test of statistical significance between subgroups: low vs middle, P = 0.64; middle vs high, P = 0.89; low vs high, P = 0.60.<br>Cessation own-price elasticity, smokeless tobacco:<br>– all: 0.003 (95% CI 0.001, 0.004)<br>Wealth:<br>- tertile 1 (low): 0.003 (95%CI 0.000, 0.005)<br>- tercile 2: 0.004 (95%CI 0.000, 0.007)<br>- tercile 3: 0.001 (95%CI -0.002, 0.004)<br>Chi-squared test of statistical significance between subgroups: low vs middle, P = 0.50; middle vs high, P = 0.42; low vs high, P = 0.70.<br>*Summary of findings*: although there were some statistically significant socioeconomic differences in price responsiveness for initiation, differences were very small and unlikely to be economically significant; no evidence of any statistically or economically significant socioeconomic differences in price responsiveness for cessation.<br>Source: Tables 3, 4, 5
Limitations	- unclear description of dependent variables;<br>- unclear how missing data were handled;<br>- no testing for misspecification;<br>- functional form of duration/time dependency not clearly reported;<br>- unclear how prices were matched to retrospective individual level data; interval to match likely very wide;<br>- survey data used did not allow to separate bidi and cigarette smoking;<br>- weighted wholesale price data used as a proxy for retail prices; no price variation in space (a single annual price for whole of India);<br>- initiation models assumed that everyone eventually failed.<br>Risk of bias: very high

Funding, competing interests reported?	Funding: Campaign for Tobacco-free Kids<br>Competing interests: disclosed (none reported)

Selvaraj S, Srivastava S, Karan A. Price elasticity of tobacco products among economic classes in India, 2011-2012. BMJ Open 2015;5(12):e008180. doi: 10.1136/bmjopen-2015-008180

Country/Journal	- India<br>- BMJ Open
Data	- Type: cross-sectional (2011-2012)<br>- Sample size: 101,662 hh<br>- Population: hh, representative at national level;<br>- Missing data: not reported;<br>- Source: National Sample Survey
Methods	Deaton's two-equation system of budget shares and unit values
Dependent variables	- Share of the budget devoted to cigarettes<br>- Share of the budget devoted to bidis<br>- Share of the budget devoted to loose leaf tobacco
Price/tax variable(s)	Unit values (expenditures/quantity consumed) at cluster-level (# of clusters not reported);<br>Deflator: unclear<br>Source: n/a
Covariates	hh expenditure, hh size, ratio of males in hh (15 years+), education, religion, social group (caste), urban/rural
Misspecification tests	Unclear
Sensitivity analyses	Unclear
Results	Total own-price elasticity, cigarettes:<br>- tercile 1, low: -0.83 (95%CI -0.84, -0.82)<br>- tercile 2, mid: -0.09 (95%CI -0.10, -0.08)<br>- tercile 3, high: -0.26 (95%CI -0.26, -0.26)<br>Total own-price elasticity, bidis:<br>- tercile 1, low: -0.43 (95%CI -0.43, -0.43)<br>- tercile 2, mid: -0.25 (95%CI -0.25, -0.25)<br>- tercile 3, high: -0.08 (95%CI -0.09, -0.07)<br>Total own-price elasticity, leaf tobacco:<br>- tercile 1, low: -0.56 (95%CI -0.56, -0.56)<br>- tercile 2, mid: -0.45 (95%CI -0.45, -0.45)<br>- tercile 3, high: -0.05 (95%CI -0.06, -0.04)<br>*Summary of findings*: estimates suggest that lower socioeconomic status household were more responsive to price; differences were large enough to be economically meaningful. These results, however, should be interpreted with caution as the reported standard errors too small to be believable.<br><br>Source: Table 3

Limittations	- unclear how missing data/outliers were dealt with;<br>- unclear adjustment for inflation;<br>- no testing for misspecification;<br>- no sensitivity analyses;<br>- # of clusters not reported; # of household with positive cigarette/bidi/tobacco leaf expenditures within each cluster not clearly reported;<br>- imprecision: reported standard errors too small to be believable;<br>- no formal assessment of socioeconomic differences in price responsiveness.<br>Risk of bias: very high
Funding, competing interests reported?	Funding: International Development Research Center (IDRC)<br>Competing interests: disclosed (none reported).

Guindon GE, Nandi A, Chaloupka FJ, et al. Socioeconomic Differences in the Impact of Smoking Tobacco and Alcohol Prices on Smoking in India. NBER Working Paper No. 17580. Cambridge, MA: National Bureau of Economic Research, 2011.

Country/Journal	- India<br>- NBER Working Paper
Data	- Type: repeated cross-sectional (1993/94; 1999/00; 2000/01; 2001/02; 2003; 2004; 2004/05 - 2007/08)<br>- Sample size: 115,354 hh; 120,309 hh; 57,273 hh; 62,628 hh; 41,013 hh; 29,631 hh; 124,644 hh; 39,438 hh; 63,729 hh; 50,297 hh (respectively)<br>- Population: hh, representative at national level;<br>- Missing data: not reported;<br>- Source: National Sample Survey (NSS)
Methods	Share-log functional form using multilevel regressions.
Dependent variables	Share of the budget devoted to cigarettes, bidis, and country liquor.
Price/tax variable(s)	Unit values (expenditures/quantity consumed) at cluster-level (districts)<br>Deflator: Consumer Price Index for Industrial Workers (CPI-IW)<br>Source: Labour Bureau
Covariates	Religion; urban/rural; share of men in hh; share of adults in hh; hh head education, sex, age; NSS round.
Misspecification tests	None
Sensitivity analyses	Estimated models using average unit values by cluster over all households (instead of just households which reported consuming cigarettes, bidis or country liquor); included quarterly and regional dummies to capture regional and temporal variations; included all outliers/excluded outliers whose logs lied more than 2.5 standard deviations from the mean of logs.

Results	– Unit values averaged by cluster over all households:<br>Own-price elasticities, bidis:<br>- All: -0.94 (95%CI -0.98, -0.90)<br>- Household total expenditures :<br>• Low: -0.95 (95%CI -0.99, -0.91)<br>• High: -0.86 (95%CI -0.94, -0.79)<br>- Education:<br>• <= primary: -0.92 (95%CI -0.96, -0.88)<br>• > primary: -0.94 (95%CI -0.99, -0.91)<br>Own-price elasticities, cigarettes:<br>- All: -1.02 (95%CI -1.08, -0.97)<br>- Household total expenditures :<br>• Low: -1.11 (95%CI -1.21, -1.02)<br>• High: -0.99 (95%CI -1.05, -0.93)<br>- Education:<br>• <= primary: -1.16 (95%CI -1.26, -1.06)<br>• > primary: -0.95 (95%CI -1.03, -0.87)<br>– Unit values averaged by cluster only over households under examination:<br>Own-price elasticities, bidis:<br>- All: -0.94 (95%CI -0.98, -0.90)<br>- Household total expenditures :<br>• Low: -0.95 (95%CI -1.01, -0.90)<br>• High: -0.89 (95%CI -0.95, -0.83)<br>- Education:<br>• <= primary: -0.91 (95%CI -0.97, -0.85)<br>• > primary: -0.93 (95%CI -0.99, -0.87)<br>Own-price elasticities, cigarettes:<br>- All: -1.02 (95%CI -1.08, -0.97)<br>- Household total expenditures :<br>• Low: -0.96 (95%CI -1.04, -0.88)<br>• High: -1.02 (95%CI -1.10, -0.94)<br>- Education:<br>• <= primary: -1.02 (95%CI -1.09, -0.94)<br>• > primary: -1.00 (95%CI -1.18, -1.02)<br>*Summary of findings*: no evidence of any statistically or economically significant socioeconomic differences in price responsiveness for cigarette or bidi smoking.<br><br>Source: Table 6
Limitations	- no testing for misspecification;<br>- unclear how missing data were dealt with;<br>- no account for measurement error or quality;<br>- no formal assessment of socioeconomic differences in price responsiveness.<br>Risk of bias: high
Funding, competing interests reported?	Funding: Bill & Melinda Gates Foundation;<br>Competing interests: not disclosed.

Raei B, Emamgholipour S, Takian A, et al. Distributional health and financial consequences of increased cigarette tax in Iran: extended cost-effectiveness analysis. Health Econ Rev 2021;11(1):30. doi: 10.1186/s13561-021-00328-w

Country/Journal	- Iran<br>- Health Economics Review
Data	- Type: repeated cross-sectional (2002-2017);<br>- Sample size: ≈ 110,00 hh per expenditure quintile;<br>- Population: hh, representative at national level;<br>- Missing data: unclear;<br>- Source: Household Income and Expenditure Survey (HIES).
Methods	Two-part model:<br>- Participation: probit (prices in ln)<br>- Consumption: OLS
Dependent variables	- Participation, positive cigarette expenditures<br>- Consumption, unclear how it was defined
Price/tax variable(s)	Unclear, likely unit values;<br>Deflator: unclear;<br>Source: not reported
Covariates	Household total expenditures; household share of members who were: aged 15-18 and ≥ 65, female aged 19 to 64; jobless, with at least primary education, university education; household head age and sex; divorce rate, unemployment rate at province-level; survey years.
Misspecification tests	None
Sensitivity analyses	None
Results	Participation own-price elasticity, cigarettes*:<br>– Household total expenditures:<br>- quintile 1 (low): -0.07 (P < 0.01)<br>- quintile 2: -0.11 (P < 0.01)<br>- quintile 3: -0.12 (P < 0.01)<br>- quintile 4: -0.12 (P < 0.01)<br>- quintile 5: -0.11 (P < 0.01)<br>Consumption own-price elasticity, cigarettes*:<br>– Household total expenditures:<br>- quintile 1 (low): -0.40 (P < 0.01)<br>- quintile 2: -0.36 (P < 0.01)<br>- quintile 3: -0.36 (P < 0.01)<br>- quintile 4: -0.37 (P < 0.01)<br>- quintile 5: -0.32 (P < 0.01)<br>* measures of uncertainty not reported<br>*Summary of findings*: no evidence of any statistically or economically significant socioeconomic differences in price responsiveness for cigarette smoking.<br><br>Source: Appendix Tables 2, 3, 4

Limitations	- dependent variable not clearly defined;<br>- unclear how missing data/outliers were handled;<br>- no description of price variable; likely unit values (clusters not defined; no. of clusters not reported; no. of unit values per cluster not reported);<br>- unclear adjustment for inflation;<br>- no testing for misspecification;<br>- no sensitivity analyses;<br>- no account for measurement error or quality (if unit values were used as proxy for prices);<br>- socioeconomic differences in price responsiveness not formally assessed.<br>Risk of bias: very high
Funding, competing interests reported?	Funding: Tehran University of Medical Sciences; Health Information Management Research Center<br>Competing interests: disclosed (none reported).

Nayab D, Nasir M, Memon JA, et al. Estimating the price elasticity for cigarette and chewed tobacco in Pakistan: evidence from microlevel data. Tob Control 2020;29(Suppl 5):s319. doi: 10.1136/tobaccocontrol-2019-055232

Country/Journal	- Pakistan<br>- Tobacco control
Data	- Type: cross-sectional (2015-16);<br>- Sample size: 24,238 hh<br>- Population: hh, representative at national level;<br>- Missing data: unclear;<br>- Source: Household Integrated Income and Consumption Survey (HIICS).
Methods	Deaton's two-equation system of budget shares and unit values
Dependent variables	- share of the budget devoted to cigarettes;<br>- share of the budget devoted to chewed tobacco (composite commodity including saunf, naswar, gutka)
Price/tax variable(s)	Unit values (expenditures/quantity consumed) at cluster-level (1087 enumeration blocks/518 villages)<br>Deflator: unclear;<br>Source: not reported
Covariates	Household total expenditures; household size; mean household education; highest degree obtained by a member of the household; education of the head of the household; share of adults in the household; share of male members in the household; no. of earners in the household; region and province of residence.
Misspecification tests	None
Sensitivity analyses	None

Results	Participation own-price elasticity, cigarettes:<br>– Household total expenditures:<br>- quintiles 1-3 (low): -1.14 (95%CI -1.35, -0.92)<br>- quintile 4-5: 0.10 (95%CI -52, 52)<br><br>Participation own-price elasticity, chewed tobacco:<br>– Household total expenditures:<br>- quintiles 1-3 (low): -0.75 (95%CI -1.08, -0.41)<br>- quintile 4-5: 0.44 (95%CI -2.7, 3.6)<br><br>*Summary of findings*: unclear if there were any statistically or economically significant socioeconomic differences in price responsiveness for cigarette smoking or chewing tobacco; estimates for higher-SES too imprecisely estimated.<br><br>Source: Table 3
Limitations	- unclear how missing data/outliers were handled;<br>- unclear adjustment for inflation;<br>- no. of unit values per cluster not reported; no. of clusters with positive cigarette/chewing tobacco expenditures not reported;<br>- no testing for misspecification;<br>- no sensitivity analyses;<br>- imprecision: uncertainty intervals very wide;<br>- socioeconomic differences in price responsiveness not formally assessed.<br><br>Risk of bias: high
Funding, competing interests reported?	Funding: Bloomberg Philanthropies; University of Illinois Chicago<br>Competing interests: disclosed (none reported)

Europe: Eastern Europe

Gjika A, Zhllima E, Imami D. Chapter 3. Albania. In: Zubović J, Vladisavljević M, eds. Impacts of Tobacco Excise Increases on Cigarette Consumption and Government Revenues in Southeastern European Countries. Chicago: Institute for Health Research and Policy, University of Illinois Chicago 2019:18-24.

Country/Journal	- Albania<br>- Report (University of Illinois Chicago)
Data	- Type: repeated cross-sectional (2014-2017)<br>- Sample size: 28,748 hh<br>- Population: hh, representative at national level;<br>- Missing data: not reported;<br>- Source: Household Budget Survey (HBS)
Methods	Two-part model:<br>- Participation: logit (prices in ln)<br>- Consumption: Deaton's two-equation system of budget shares and unit values; generalized linear model (GLM)
Dependent variables	- Participation: not clearly reported; likely positive household expenditures on cigarettes vs. no expenditures on cigarettes.<br>- Consumption: share of the budget devoted to cigarettes.
Price/tax variable(s)	Unit values (expenditures/quantity consumed) at cluster-level (cluster not defined); no. of clusters not reported;<br>Deflator: unclear<br>Source: unclear
Covariates	Not clearly reported; total expenditures; "share of men and adults in the household, maximum or mean level of education and activity of the household members), region and settlement fixed effects and variables representing institutional changes relevant to cigarette consumption."
Misspecification tests	None
Sensitivity analyses	Generalized Linear Model (GLM)
Results	Participation own-price elasticity, cigarettes:<br>– all: -0.17 (95%CI -0.34, 0.01)<br>Household total expenditures<br>- Low: -0.92 (95%CI -1.40, -0.44)<br>- Middle: -0.23 (95%CI -0.52, 0.06)<br>- High: -0.35 (95%CI -0.69, -0.02)<br>Consumption own-price elasticity, cigarettes*:<br>– all: -0.27 (95%CI -0.46, -0.07)<br>Household total expenditures:<br>- Low: -0.28 (95%CI -0.55, -0.01)<br>- Middle: -0.15 (95% CI -0.39, 0.09)<br>- High: -0.36 (95%CI -0.62, -0.10)<br>* results obtained using generalized linear models. Results using Deaton's approach not presented.<br>*Summary of findings*: point estimates suggest that lower socioeconomic status households were more responsive to price for smoking participation; differences were large enough to be economically significant but may not be statistically significant. No evidence that lower socioeconomic status households were more responsive to price for consumption.<br><br>Source: Table 3.5

Limitations	- unclear description of dependent variables;<br>- covariates not clearly presented;<br>- unclear how missing data/outliers were handled;<br>- unclear adjustment for inflation;<br>- clusters not defined; no. of clusters not reported; no. of household with positive cigarette expenditures within each cluster not reported;<br>- unit values treated as market prices in participation component of two-part model; unclear why Deaton's two-equation system was not used to obtain total own-price elasticities; Results using Deaton's approach not presented for price elasticity estimates by SES categories;<br>- no testing for misspecification;<br>- imprecision: uncertainty intervals very wide;<br>- selective reporting of results;<br>- no formal assessment of socioeconomic differences in price responsiveness.<br>Risk of bias: very high
Funding, competing interests reported?	Funding: Bloomberg Philanthropies; University of Illinois Chicago<br>Competing interests: not disclosed

Gligoric D, Preradovic Kulovac D, Micic L, et al. Price and income elasticity of cigarette demand in Bosnia and Herzegovina by different socioeconomic groups. Tob Control 2022 doi: 10.1136/tobaccocontrol-2021-056881

Country/Journal	- Bosnia and Herzegovina<br>- Tobacco Control
Data	- Type: repeated cross-sectional (2007, 2011, 2015)<br>- Sample size: 21,424 hh; 9,953 smoking hh<br>- Population: hh, representative at national level;<br>- Missing data: not reported;<br>- Source: Household Budget Survey (HBS)
Methods	Two-part model:<br>- Participation: logit (prices in ln)<br>- Consumption: Deaton's two-equation system of budget shares and unit values
Dependent variables	- Participation: not clearly reported; likely positive household expenditures on cigarettes vs. no expenditures on cigarettes.<br>- Consumption: share of the budget devoted to cigarettes.
Price/tax variable(s)	Unit values (expenditures/quantity consumed) at cluster-level (municipality); 9908 smoking households nested in 389 clusters;<br>Deflator: Consumer Price Index<br>Source: unclear
Covariates	Total monthly expenditures; household size; age; sex; mean and max education level of household members (unclear how it was operationalized); household adult and male ratio; urban/rural; household type (employed, self-employed, pensioner, unemployed).
Misspecification tests	Link test; Hosmer and Lemeshow goodness of fit test;
Sensitivity analyses	None

Results	Participation own-price elasticity, cigarettes:<br>– all: -0.55 (95% CI -0.65, -0.45)<br>Household total expenditures<br>- Low: -0.80 (95%CI -0.88, -0.71)<br>- Middle: -0.54 (95%CI -0.62, -0.46)<br>- High: -0.33 (95%CI -0.41, -0.25)<br>Chi-squared tests of statistical significance between subgroups: P < 0.01 for all comparisons.<br>Consumption own-price elasticity, cigarettes:<br>– all: -0.46 (95% CI -0.53, -0.39)<br>Household total expenditures:<br>- Low: -0.61 (95%CI -0.70, -0.51)<br>- Middle: -0.45 (95%CI -0.55, -0.36)<br>- High: -0.37 (95%CI -0.49, -0.24)<br>Chi-squared test of statistical significance between subgroups: low vs middle, P = 0.01; middle vs high, P = 0.26; low vs high, P < 0.01.<br>Total own-price elasticity, cigarettes:<br>– all: -1.01 (95%CI -1.10, -0.93)<br>Household total expenditures:<br>- Low: -1.40 (95% CI -1.52, -1.28)<br>- Middle: -0.99 (standard errors not reported, P < 0.01)<br>- High: -0.67 (95%CI -0.82,-0.52)<br>Chi-squared tests of statistical significance between subgroups: P < 0.01 for all comparisons.<br>*Summary of findings*: point estimates suggest that lower socioeconomic status households were more responsive to price; differences were large enough to be economically significant.<br><br>Source: Table 5
Limitations	- unclear how missing data were handled;<br>- no sensitivity analyses;<br>- unit values treated as market prices in participation component of two-part model; unclear why Deaton's two-equation system was not used to obtain total own-price elasticities.<br>Risk of bias: moderate
Funding, competing interests reported?	Funding: University of Illinois at Chicago's Institute for Health Research and Policy; Bloomberg Philanthropies<br>Competing interests: disclosed (none reported)

Prekazi B, Pula E. Chapter 5. Kosovo. In: Zubović J, Vladisavljević M, eds. Impacts of Tobacco Excise Increases on Cigarette Consumption and Government Revenues in Southeastern European Countries. Chicago: Institute for Health Research and Policy, University of Illinois Chicago 2019:34-41.

Palushi L, Prekazi B, Statovci J, et al. Accelerating Progress on Effective Tobacco Tax Policies in Low- and Middle-Income Countries. National Study - Kosovo. Pristina: Centre for Political Courage, 2018.

Country/Journal	- Kosovo<br>- Report (University of Illinois Chicago)

Data	- Type: repeated cross-sectional (2007-2017)<br>- Sample size: 26,311 hh nested in 341 clusters; 10,217 households with positive cigarette expenditure<br>- Population: hh, representative at national level;<br>- Missing data: not reported;<br>- Source: Household Budget Survey (HBS)
Methods	Two-part model:<br>- Participation: logit (prices in ln)<br>- Consumption: Deaton's two-equation system of budget shares and unit values; generalized linear model (GLM)
Dependent variables	- Participation: positive household expenditures on cigarettes vs. no expenditures on cigarettes.<br>- Consumption: share of the budget devoted to cigarettes.
Price/tax variable(s)	Unit values (expenditures/quantity consumed) at cluster-level (38 municipalities);<br>Deflator: unclear<br>Source: unclear
Covariates	Not clearly reported; total expenditures; "share of men and adults in the household, maximum or mean level of education and activity of the household members), region and settlement fixed effects and variables representing institutional changes relevant to cigarette consumption."
Misspecification tests	None
Sensitivity analyses	Generalized Linear Model (GLM)
Results	Participation own-price elasticity, cigarettes:<br>– all: -0.12 (95%CI -2.24, 1.99)<br>Household total expenditures<br>- Low: -0.66 (95%CI -2.44, 1.12)<br>- Middle: 0.00 (95%CI -2.10, 2.11)<br>- High: -0.47 (95%CI -2.88, 1.95)<br>Consumption own-price elasticity, cigarettes*:<br>– all: -0.39 (95% CI -0.63, -0.15)<br>Household total expenditures:<br>- Low: -0.53 (95%CI -0.95, -0.11)<br>- Middle: -0.63 (95%CI -1.13, -0.13)<br>- High: -0.29 (95%CI -1.25, 0.66)<br>* results obtained using generalized linear models. Results using Deaton's approach not presented.<br>*Summary of findings*: no evidence that lower socioeconomic status households were more responsive to price.<br><br>Source: Table 5.5
Limitations	- covariates not clearly presented;<br>- unclear how missing data/outliers were handled;<br>- unclear adjustment for inflation;<br>- unit values treated as market prices in participation component of two-part model; unclear why Deaton's two-equation system was not used to obtain total own-price elasticities; Results using Deaton's approach not presented for price elasticity estimates by SES categories;<br>- no testing for misspecification;<br>- imprecision: uncertainty intervals very wide;<br>- no formal assessment of socioeconomic differences in price responsiveness.<br>Risk of bias: very high

Funding, competing interests reported?	Funding: Bloomberg Philanthropies; University of Illinois Chicago<br>Competing interests: not disclosed

Cizmovic M, Mugosa A, Kovacevic M, et al. Effectiveness of tax policy changes in Montenegro: smoking behaviour by socio-economic status. Tob Control 2022 doi: 10.1136/tobaccocontrol-2021-056876

Country/Journal	- Montenegro<br>- Tobacco Control
Data	- Type: repeated cross-sectional (2006-2015, 2017)<br>- Sample size: 12,503 hh; 5793 smoking households<br>- Population: hh, representative at national level;<br>- Missing data: not reported;<br>- Source: Household Budget Survey (HBS)
Methods	Two-part model:<br>- Participation: logit (prices in ln)<br>- Consumption: Deaton's two-equation system of budget shares and unit values; generalized linear models.
Dependent variables	- Participation: positive household monthly expenditures on cigarettes vs. no expenditures on cigarettes.<br>- Consumption: share of the budget devoted to cigarettes.
Price/tax variable(s)	Unit values (expenditures/quantity consumed) at cluster-level (municipality); 5793 smoking households nested in 518 clusters;<br>Deflator: Consumer Price Index<br>Source: unclear
Covariates	Total monthly expenditures; household size; adult and male ratio in household; highest education level among household members; household type (employed, pensioner, unemployed).
Misspecification tests	Link tests; Hosmer and Lemeshow goodness of fit test; test for multicollinearity.
Sensitivity analyses	Alternative functional for price and income were used; preferred model chosen based on Akaike information criterion (AIC), Bayesian information criterion (BIC) and log-likelihood. Results not reported; generalized linear models were estimated in addition to Deaton's two-equation system of budget shares and unit values.

Results	Participation own-price elasticity, cigarettes:<br>– all: -0.52 (95%CI -0.60, -0.44)<br>Household total expenditures:<br>- Low: -0.60 (95%CI -0.73, -0.46)<br>- Middle: -0.58 (95%CI -0.71, -0.46)<br>- High: -0.34 (95%CI -0.49, -0.20)<br>Chi-squared tests of statistical significance between subgroups: low vs middle, P = 0.77; middle vs high, P < 0.01; low vs high, P < 0.01.<br>Consumption own-price elasticity, cigarettes:<br>– all: -0.36 (95% CI -0.45, -0.27)<br>Household total expenditures:<br>- Low: -0.42 (95% CI -0.52, -0.33)<br>- Middle: -0.34 (95%CI -0.47, -0.21)<br>- High: -0.26 (95%CI -0.54, 0.02)<br>Chi-squared tests of statistical significance between subgroups: P > 0.20 for all comparisons.<br>Total own-price elasticity, cigarettes:<br>– all: -0.88 (95%CI -0.99, -0.77)<br>Household total expenditures:<br>- Low: -1.02 (95%CI -1.18, -0.85)<br>- Middle: -0.92 (95%CI -1.11, -0.74)<br>- High: -0.60 (95%CI -0.89, -0.31)<br>Chi-squared tests of statistical significance between subgroups: low vs middle, P = 0.22; middle vs high, P < 0.01; low vs high, P < 0.01.<br><br>*Summary of findings*: estimates suggest that low and middle socioeconomic status households were more responsive to price; differences were large enough to be economically meaningful. Socioeconomic differences in price responsiveness were driven by differences in own-price participation elasticities.<br><br>Source: Tables 3, 4, 5
Limitations	- unclear how missing data were handled;<br>- unit values treated as market prices in participation component of two-part model; unclear why Deaton's two-equation system was not used to obtain total own-price elasticities;<br>- relatively few clusters per survey year (≈ 15).<br>Risk of bias: high
Funding, competing interests reported?	Funding: University of Illinois at Chicago's Institute for Health Research and Policy; Bloomberg Philanthropies<br>Competing interests: disclosed (none reported)

Najdova N. Chapter 6. North Macedonia. In: Zubović J, Vladisavljević M, eds. Impacts of Tobacco Excise Increases on Cigarette Consumption and Government Revenues in Southeastern European Countries. Chicago: Institute for Health Research and Policy, University of Illinois Chicago 2019:42-50.

Country/Journal	- Kosovo<br>- Report (University of Illinois Chicago)
Data	- Type: repeated cross-sectional (2015-2017)<br>- Sample size: 8,593<br>- Population: hh, representative at national level;<br>- Missing data: not reported;<br>- Source: Household Budget Survey (HBS)

Methods	Two-part model:<br>- Participation: logit (prices in ln)<br>- Consumption: Deaton's two-equation system of budget shares and unit values; generalized linear model (GLM)
Dependent variables	- Participation: positive household expenditures on cigarettes vs. no expenditures on cigarettes.<br>- Consumption: share of the budget devoted to cigarettes.
Price/tax variable(s)	Unit values (expenditures/quantity consumed) at cluster-level (clusters not defined); no. of clusters not reported;<br>Deflator: unclear<br>Source: unclear
Covariates	Not clearly reported; total expenditures; "share of men and adults in the household, maximum or mean level of education and activity of the household members), region and settlement fixed effects and variables representing institutional changes relevant to cigarette consumption."
Misspecification tests	None
Sensitivity analyses	Generalized Linear Model (GLM)
Results	Participation own-price elasticity, cigarettes:<br>– all: -0.21 (95%CI -0.46, 0.03)<br>Household expenditures<br>- low: -0.45 (95%CI -0.92, 0.03)<br>- mid: -0.50 (95%CI -0.93, -0.06)<br>- high: 0.19 (95% CI -0.17, 0.55)<br>Consumption own-price elasticity, cigarettes:<br>– all: -0.23 (95%CI -0.28, -0.18)<br>Household expenditures:<br>- low: 0.58 (95%CI -0.20, 1.37)<br>- mid: -0.44 (95%CI -1.46, 0.57)<br>- high: -0.28 (95%CI -1.06, 0.50)<br>*Summary of findings*: point estimates suggest that higher socioeconomic status households were less responsive to price for smoking participation; it is unclear if consumption own-price elasticities varied by socioeconomic status.<br><br>Source: Table 6.4
Limitations	- covariates not clearly presented;<br>- unclear how missing data/outliers were handled;<br>- unclear adjustment for inflation;<br>- unit values treated as market prices in participation component of two-part model; unclear why Deaton's two-equation system was not used to obtain total own-price elasticities;<br>- no. of clusters not reported;<br>- no testing for misspecification;<br>- imprecision: uncertainty intervals very wide;<br>- no formal assessment of socioeconomic differences in price responsiveness.<br>Risk of bias: very high
Funding, competing interests reported?	Funding: Bloomberg Philanthropies; University of Illinois Chicago<br>Competing interests: not disclosed

Vladisavljević M, Đukić M, Jovanović O, et al. Chapter 8. Serbia. In: Zubović J, Vladisavljević M, eds. Impacts of Tobacco Excise Increases on Cigarette Consumption and Government Revenues in Southeastern European Countries. Chicago: Institute for Health Research and Policy, University of Illinois Chicago 2019:60-69.

Vladisavljevic M, Zubovic J, Dukic M, et al. Tobacco price elasticity in Serbia: evidence from a middle-income country with high prevalence and low tobacco prices. Tob Control 2020;29(Suppl 5):s331-s36. doi: 10.1136/tobaccocontrol-2019-055262

Country/Journal	- Serbia<br>- Report (University of Illinois Chicago)
Data	- Type: repeated cross-sectional (2006-2017)<br>- Sample size: 61,889 hh; 39% had monthly expenses on cigarettes;<br>- Population: hh, representative at national level;<br>- Missing data: not reported;<br>- Source: Household Budget Survey (HBS)
Methods	Two-part model:<br>- Participation: logit (prices in ln)<br>- Consumption: Deaton's two-equation system of budget shares and unit values; generalized linear model (GLM)
Dependent variables	- Participation: positive household expenditures on cigarettes vs. no expenditures on cigarettes.<br>- Consumption: share of the budget devoted to cigarettes.
Price/tax variable(s)	Unit values (expenditures/quantity consumed) at cluster-level (clusters not defined); no. of clusters not reported; Vladisavljevic, Zubovic et al., 2020 reports about 150 'municipalities' per survey cycle.<br>Deflator: unclear; Vladisavljevic, Zubovic et al., 2020 reports CPI<br>Source: unclear; Vladisavljevic, Zubovic et al., 2020 reports Statistical Office of the Republic of Serbia.
Covariates	Not clearly reported; total expenditures; "share of men and adults in the household, maximum or mean level of education and activity of the household members), region and settlement fixed effects and variables representing institutional changes relevant to cigarette consumption."<br>Vladisavljevic, Zubovic et al., 2020 reports total expenditures; household size; urban/rural; age; sex composition of the household; mean and maximum level of education of the household members; household type (employed, self-employed, pensioner, unemployed).
Misspecification tests	None
Sensitivity analyses	Generalized Linear Model (GLM)

Results	Participation own-price elasticity, cigarettes:<br>– all: -0.27 (95%CI -0.36, -0.17)<br>Household expenditures<br>- low: -0.57 (95%CI -0.71, -0.42)<br>- mid: -0.26 (95%CI -0.40, -0.12)<br>- high: -0.04 (95%CI -0.17, 0.09)<br>Consumption own-price elasticity, cigarettes:<br>– all: -0.40 (95%CI -0.50, -0.29)<br>Household expenditures:<br>- low: -0.51 (95%CI -0.65, -0.38)<br>- mid: -0.37 (95%CI -0.50, -0.24)<br>- high: -0.22 (95%CI -0.30, -0.14)<br>*Summary of findings*: Point estimates suggest that lower socioeconomic status households were less responsive to price for smoking participation and consumption.<br>No formal assessment of socioeconomic differences in price responsiveness. Uncertainty intervals suggest that differences were statistically significant. Differences were likely large enough to be economically significant.<br><br>Source: Table 8.3
Limitations	- unclear how missing data/outliers were handled;<br>- unit values treated as market prices in participation component of two-part model; unclear why Deaton's two-equation system was not used to obtain total own-price elasticities;<br>- no. of clusters not clearly reported;<br>- no testing for misspecification;<br>- imprecision: uncertainty intervals fairly wide;<br>- no formal assessment of socioeconomic differences in price responsiveness.<br>Risk of bias: high
Funding, competing interests reported?	Funding: Bloomberg Philanthropies; University of Illinois Chicago<br>Competing interests: disclosed (none reported)
