# Supplementary material for: Socioeconomic differences in the impact of prices and taxes on tobacco use in low- and middle-income countries–A systematic review
Source: PLOS Glob Public Health. 2023 Sep 27;3(9):e0002342. doi: 10.1371/journal.pgph.0002342 (PMC10529577; doi:10.1371/journal.pgph.0002342)
Supplement: S4 Appendix — (PDF) [file pgph.0002342.s005.pdf]

S4 Appendix. Excluded Studies

Price effects not examined by socioeconomic status (n=10)

Ahmed, M.U., Pulok, M.H., Hashmi, R., Hajizadeh, M., & Nargis, N. (2022). Price and Income Elasticities of Cigarette Smoking Demand in Bangladesh: Evidence from Urban Adolescents and Young Adults. Nicotine Tob Res, 24, 826-833.

Boachie, M.K., Immurana, M., Agyemang, J.K., & Ross, H. (2022). Cigarette Prices and Smoking Experimentation in Sierra Leone: An Exploratory Study. Tob Use Insights, 15.

Boachie, M.K., Immurana, M., Tingum, E.N., Mdege, N.D., & Ross, H. (2022). Effect of relative income price on smoking initiation among adolescents in Ghana: evidence from pseudo-longitudinal data. BMJ Open, 12, e054367.

Gjika, A., Zhllima, E., Rama, K., & Imami, D. (2020). Analysis of Tobacco Price Elasticity in Albania Using Household Level Data. International Journal of Environmental Research and Public Health, 17.

Hu, X., Wang, Y., Huang, J., & Zheng, R. (2019). Cigarette Affordability and Cigarette Consumption among Adult and Elderly Chinese Smokers: Evidence from A Longitudinal Study. International Journal of Environmental Research and Public Health, 16.

Husain, M.J., Kostova, D., Mbulo, L., Benjakul, S., Kengganpanich, M., & Andes, L. (2017). Changes in cigarette prices, affordability, and brand-tier consumption after a tobacco tax increase in Thailand: Evidence from the Global Adult Tobacco Surveys, 2009 and 2011. Preventive Medicine, 105S, S4-S9.

Leinsalu, M., Stickley, A., & Kunst, A.E. (2015). Reduced affordability of cigarettes and socio-economic inequalities in smoking continuation in Stakhanov, Ukraine, 2009. European Journal of Public Health, 25, 216-218.

Palushi, L., Prekazi, B., Statovci, J., & Bylykbashi, N.D. (2018). Accelerating Progress on Effective Tobacco Tax Policies in Low- and Middle-Income Countries. National Study - Kosovo. Pristina: Centre for Political Courage.

Shang, C., Chaloupka, F.J., Gupta, P.C., Pednekar, M.S., & Fong, G.T. (2019). Association between tobacco prices and smoking onset: evidence from the TCP India Survey. Tobacco Control, 28, s3-s8.

Vladisavljevic, M., Zubovic, J., Dukic, M., & Jovanovic, O. (2020). Tobacco price elasticity in Serbia: evidence from a middle-income country with high prevalence and low tobacco prices. Tobacco Control, 29, s331-s336.

Too little methodological information provided (n=10)

Fuchs, A., & Del Carmen, G. (2018). The Distributional Effects of Tobacco Taxation The Evidence of White and Clove Cigarettes in Indonesia. Policy Research Working Paper 8558. Washington, DC: The World Bank.

Fuchs, A., Icaza, F.G., & Paz, D. (2019). Distributional Effects of Tobacco Taxation: A Comparative Analysis. Policy Research Working Paper 8805. Washington, DC: The World Bank.

Fuchs, A., Del Carmen, G., & Mukon, A.K. (2018). Long-Run Impacts of Increasing Tobacco Taxes. Evidence from South Africa. Washington, DC: The World Bank.

Fuchs, A., Matytsin, M., & Obukhova, O. (2018). Tobacco Taxation Incidence Evidence from the Russian Federation. Policy Research Working Paper 8626. Washington, DC: The World Bank.

Fuchs, A., & Meneses, F. (2018). Tobacco Price Elasticity and Tax Progressivity in Moldova. Policy Research Working Paper No. 8327. Washington, DC: The World Bank.

Fuchs, A., Orlic, E., & Cancho, C.A. (2019). Time to Quit: The Tobacco Tax Increase and Household Welfare in Bosnia and Herzegovina. Washington, DC: The World Bank.

Fuchs Tarlovsky, A., & González Icaza, M.F. (2020). Taxing Tobacco in Georgia. Welfare and Distributional Gains of Smoking Cessation. Policy Research Working Paper 9130. Washington, DC: The World Bank.

Postolovska, I., Lavado, R., Tarr, G., & Verguet, S. (2017). Estimating the distributional impact of increasing taxes on tobacco products in Armenia. Results from an extended cost-effectiveness analysis. Washhington, DC: The World Bank.

Postolovska, I., Nguyen, H.T.H., Sargaldakova, A., & Lavado, R. (2018). An extended cost-effectiveness analysis of tobacco price increases in the Kyrgyz Republic. Washington, DC: The World Bank.

Salti, N., Brouwer, E., & Verguet, S. (2016). The health, financial and distributional consequences of increases in the tobacco excise tax among smokers in Lebanon. Social Science and Medicine, 170, 161-169.

Not a low- or middle-income country (n=3)

Alghamdi, A., Fallatah, A., Okal, F., Felemban, T., Eldigire, M., & Almodaimegh, H. (2020). Smoking behaviour after enforcement of a 100% tax on tobacco products in Saudi Arabia: a cross-sectional study. East Mediterr Health J, 26, 39-46.

Al-Tannir M, Abu-Shaheen A, Altannir Y, Altannir M. Tobacco price increase and consumption behaviour among male smokers in Saudi Arabia: a community-based study. Eastern Mediterranean Health Journal. 2020;26(12):1518-24.

Barać, Ž.A., Burnać, P., Rogošić, A., Šodan, S., & Vuko, T. (2021). Cigarette price elasticity in Croatia – analysis of household budget surveys. Journal of Applied Economics, 24, 318-328.

Price effects not estimated (n=3)

Denisova, I., & Kuznetsova, P. (2014). The Effects of Tobacco Taxes on Health: An Analysis of the Effects by Income Quintile and Gender in Kazakhstan, the Russian Federation, and Ukraine. Health, Nutrition, and Population (HNP) Discussion Paper No. 92765. Washington, DC: The World Bank.

Fuchs, A., & Meneses, F. (2017). Regressive or Progressive? The Effect of Tobacco Taxes in Ukraine. Policy Research Working Paper No. 8227. Washington, DC The World Bank.

Fuchs, A., & Meneses, F.J. (2017). Are tobacco taxes really regressive? evidence from Chile. Washington, DC: The World Bank.

Cannot disentangle effect of price/tax change from other policies (n=1)

Tingum, E.N., Mukong, A.K., & Mdege, N. (2020). The effects of price and non-price policies on cigarette consumption in South Africa. Tobacco Induced Diseases, 18, 62.

Examined self-reported changes in smoking in response to a tax increase (n=1)

Yu, L., Cohen, J.E., Hoe, C., Yang, T., & Wu, D. (2020). Male smoking reduction behaviour in response to China's 2015 cigarette tax increase. Tobacco Control, 29, 405-411.

Main findings included in more recent publication (n=2)

Kidane, A., Mduma, J., Naho, A., Ngeh, E.T., & Hu, T.-w. (2015). The Demand for Cigarettes in Tanzania and Implications for Tobacco Taxation Policy. Advances in Economics and Business, 3, 428-435.

Nayab, D., Nasir, M., Memon, J.A., Khalid, M., & Hussain, A. (2018). Economics of Tobacco Taxation and Consumption in Pakistan. Islamabad: Pakistan Institute of Development Economics.

Outcome not tobacco use (n=1)

Kostova, D., Andes, L., Erguder, T., Yurekli, A., Keskinkilic, B., Polat, S., et al. (2014). Cigarette prices and smoking prevalence after a tobacco tax increase – Turkey, 2008 and 2012. MMWR: Morbidity and Mortality Weekly Report, 63, 457-461.

Price effects not reported (n=1)

Nargis, N., Yong, H.-H., Driezen, P., Mbulo, L., Zhao, L., Fong, G.T., et al. (2019). Socioeconomic patterns of smoking cessation behavior in low and middle-income countries: Emerging evidence from the Global Adult Tobacco Surveys and International Tobacco Control Surveys. PloS One, 14, e0220223.
