# Supplementary material for: Socioeconomic differences in the impact of prices and taxes on tobacco use in low- and middle-income countries–A systematic review
Source: PLOS Glob Public Health. 2023 Sep 27;3(9):e0002342. doi: 10.1371/journal.pgph.0002342 (PMC10529577; doi:10.1371/journal.pgph.0002342)
Supplement: Attachment — Submitted filename: review_price-tax_response_Jun2023.docx [file pgph.0002342.s006.docx]

**Socioeconomic differences in the impact of prices and taxes on tobacco use in low- and middle-income countries – a systematic review**

**Reviewers' comments:**

Reviewer's Responses to Questions

Comments to the Author

1. Does this manuscript meet PLOS Global Public Health’s publication criteria? Is the manuscript technically sound, and do the data support the conclusions? The manuscript must describe methodologically and ethically rigorous research with conclusions that are appropriately drawn based on the data presented.

Reviewer #1: Yes

Reviewer #2: Yes

2. Has the statistical analysis been performed appropriately and rigorously?

Reviewer #1: Yes

Reviewer #2: Yes

3. Have the authors made all data underlying the findings in their manuscript fully available (please refer to the Data Availability Statement at the start of the manuscript PDF file)?

The PLOS Data policy requires authors to make all data underlying the findings described in their manuscript fully available without restriction, with rare exception. The data should be provided as part of the manuscript or its supporting information, or deposited to a public repository. For example, in addition to summary statistics, the data points behind means, medians and variance measures should be available. If there are restrictions on publicly sharing data—e.g. participant privacy or use of data from a third party—those must be specified.

Reviewer #1: Yes

Reviewer #2: Yes

4. Is the manuscript presented in an intelligible fashion and written in standard English?

PLOS Global Public Health does not copyedit accepted manuscripts, so the language in submitted articles must be clear, correct, and unambiguous. Any typographical or grammatical errors should be corrected at revision, so please note any specific errors here.

Reviewer #1: Yes

Reviewer #2: Yes

5. Review Comments to the Author

Please use the space provided to explain your answers to the questions above. You may also include additional comments for the author, including concerns about dual publication, research ethics, or publication ethics. (Please upload your review as an attachment if it exceeds 20,000 characters)

Reviewer #1: I have three comments.

1. I find the description of the rationale for the review unsatisfactory. As stated by the authors in the Methods section, "Studies that examine associations between prices, taxes and tobacco use often use methodological approaches that are overlooked in risk of bias assessment tools. ... Due to heterogeneity between studies, a meta-analysis was not possible." I believe the reader would like to see a discussion on the possible explanations for the choice of the methodological approach and the existence of such heterogeneity among the studies. This is related to comment 2.

We now provide more details as to why we felt it was not possible to conduct a meta-analysis.

2. What is the objective of the review? As stated by the authors, "Our objective was to systematically review quantitative studies that examined if socioeconomic status modified the association between prices and taxes and tobacco use in LMICs." Review the quantitative studies for what purpose? Evaluate the final result? The methodological issues? What are the problems in this field? Insufficient data? Insufficient methodological knowledge by the researchers? This is related to comment 3.

We have added a short section that more clearly details the rationale and objective of our review. In very short, to properly assess the benefits and costs of higher tobacco taxes, one needs information about how about individuals respond when faced with higher taxes and prices.

3. I would like to see a more informative discussion regarding the implications for both policy and future research on the issue. It is stated in the Conclusions section that "The lack of evidence of socioeconomic differences in tobacco price responsiveness suggests that tobacco tax increases may disproportionally affect poorer individuals if they are not more responsive to increases in tobacco prices than richer individuals.”

We’ve extended our policy implication discussion. We now more clearly stress that the focus of policies should not overly focus on the ability to pay, but rather on the overall welfare effects, including, at the very least, the effect on health.

The paper's contribution is not so clear. The analysis in the review makes me think that it is the data and mainly methodological problems that drive the results in the reviewed studies. So, I would like to see some recommendations on how to conduct future research on the matter as well as some discussion on the real added value of all these published studies!

We agree that the data and methodological problems drive the results of our review. In the discussion section, we had highlighted a number of data and methodological limitations in existing studies (paragraphs 2-4). We’ve added more concrete recommendations for future research.

Reviewer #2: Thank you for the opportunity to review this interesting paper conducting a systematic review of the literature that considers whether low SES tobacco users in LMIC are more price sensitive. I found the study to be interesting and the topic is certainly of broad interest to the tobacco control community given the importance placed on tobacco tax increases. I found the study to have been well conducted and hence I think it could make a real and important contribution to the literature although I would like to see some changes made first. In particular I think the study suffers from a lack of clarity and information at various points, particularly in terms of methodology and the issue of how bias is identified in the studies highlighted. The specific comments below provide details on my thoughts at relevant points in the paper.
Good luck with the revisions!

Specific points

P.2, the abstract. The 2nd to 4th lines need a little bit more nuance, as I had to read the lines a few times to understand the points being made – I initially thought the wording contradicted itself. I would slightly reword to explain how the tax can be regressive. I also think the need for the review could be more strongly articulated, including why only LMIC were considered – there is nothing about what is already known about the issue in high income countries. That might usefully set the scene and hence the knowledge gap to be investigated.

We did our best to address the issues raised given the space constraint.

P.4, last paragraph of the introduction. I think there could be a bit more justification of the study. The final paragraph is quite blunt in that it says what the aim of the study is but doesn’t explicitly link that back to the literature review mentioned above and hence a need. Furthermore, that literature review only considers two large studies, firstly from the Word Bank and then the IARC review – I think it would be good to establish clearly that there are currently no academic reviews conducted of work in this area, and also that further country-level studies have been conducted since the IARC report was published and hence there is a need for such a review.

We’ve added a clearer rationale for conducting our review. We also now briefly discuss academic reviews.

P.4, methodology. Why did your review only start on 1st Jan 2010? To do so basically means you will be ignoring most, if not all of the literature reviewed in the IARC study which could risk bias. I had expected that the review would consider all work in this area, not just the most recent work. As such I think you need to justify your start timing, even if it is just do so because of practical reasons. I see you return to this later on in page 5 but I do think the impact of this needs to be discussed.

We did not ignore studies published before 2010. We devote a fair amount of space to them in the introduction and have extracted detailed results for each study (see Table A1). We started in 2010 because of the comprehensive review conducted by IARC. We didn’t expect to find enough studies missed by IARC that would alter the weak evidence base presented in the IARC report. We’ve tried to make this point clearer.

P.7, first paragraph. Why was the review protocol not registered? That is often done with such studies. Furthermore, such studies often adopt the PRISMA guidelines for conducting the study but that isn’t mentioned (although I see there is a check-list linked at the end of the proof). What seems to have been done is in line with those guidelines so I think it would help the reader gain some confidence if they could be explicitly referred to in the methodology section.

With hindsight, we should have posted our protocol on a website like osf.io (Open Science Foundation), as PROSPERO does not typically accept protocols that do not have a health outcome (e.g., we had a protocol rejected that proposed to examine the impact of the FCTC on policy changes [primary outcome] and tobacco use/health [secondary outcomes]).

Note that the key components of our protocol were part of an IDRC grant proposal. Without diminishing the importance of pre-registered review protocols, we feel there is nothing controversial in how we conducted our review. Our search was comprehensive, our quality assessment comprehensive and transparent. Additionally, unlike many reviews that only provide yes/no or scores, we documented reasons behind all our assessments. Lastly, we did not exclude any studies that met our inclusion criteria because of poor quality and provide a list of excluded studies and reason for exclusions.

P.8, final paragraph of section before study findings. When identifying the funding sources I found the wording a touch cumbersome and think it would have been better to say 13 studies identified Bloomberg funding, and nine identified IDRC funding.

Edited.

P.8/9, findings. You report on the likelihood of bias in the various studies identified as showing results that indicate a possible SES impact. However, you don’t qualify the direction of the possible bias – it could be that the bias leads to either over or under-reporting of relevant results. That is critical as it speaks to the overall likelihood of studies generating findings of an impact that is real and meaningful in LMIC market contexts. Furthermore, the risk of bias doesn’t in fact mean that the studies were biased – as table 1 shows clearly, most of the issues you highlight are a lack of information being reported rather than something done that is concerning in and of itself.

As we describe in the method section, when assessing the overall risk of bias of individual studies, we did not “add up” elements. Rather, we assessed each study holistically by looking at all elements. Although unclear reporting may be a signal for poor execution, more weight was put on our assessment of the methodological approaches. Also note that we did not assess any studies at serious or critical risk of bias based solely on unclear reporting. We tried to make this clearer in the method section. We do not agree that the main issue for most studies was poor reporting.

Regarding the direction of the bias, it very difficult to comment on as our objective is to examine differences in own-price effects between SES categories. Even if when we can comment on the direction of the bias for total price effects (as we did when discussing measurement error and quality), it is difficult to comment on bias working differently across SES categories. On the whole, we feel that poor data/methods likely yield estimates that are more imprecise, and hence, less likely to be different between SES categories.

We now discuss this issue in the limitation section.

P.9, 4th paragraph. You present a long list of studies that provide no evidence of SES but don’t comment as to whether any or all of these studies were assessed as being possibly biased or not. Given bias can impact a null result too, I think it is important to be explicit in regards to these studies.

Good point. We now provide our risk of bias assessment as we present these null results.

P.9, 5th paragraph. You mention studies looking at tobacco products beyond cigarettes. Did these studies consider multiple tobacco products or were they only concentrated on such particular non-tobacco products. If they do the latter, the studies might in themselves be biased as such non cigarette products are very much used by low SES groups and hence there will inevitably be a bias sample.

Table 1 describes all products examined, while Table 2 presents results for all products. A short summary:

Bangladesh: bidi, cigarette

India: bidi, cigarette

India: bidi, cigarette, leaf tobacco

India: bidi/cigarette (combined into a single measure)

Pakistan: cigarette, chewed tobacco (composite commodity including saunf, naswar, gutka)

Thailand: cigarette, roll-your-own

Bidis, for example, are definitely more popular among low-SES individuals in southern Asia. But we’re not sure in what way higher bidi use among low-SES would bias price effects.

P.10, 2nd paragraph of Risk of bias section. As with my comment above I didn’t like the way your wording joined together the description of the dependent and independent variables. Better to separate the wording of these two, i.e. Nine studies did not provide a clear description of the dependent variable while seven did not do so for the independent variable.

Fixed.

P.12, limitations. This feels a bit of an add on rather than a full consideration of the limits of the study herein. I would encourage the authors to expand this to include some of the issues I have outlined above. E.g. what does the lack of the study protocol being published in advance actually mean in practice?

We’ve extended the limitation section. Regarding publishing the review protocol in advance, as mentioned above, we can’t think of any meaningful deviation from a protocol we could have made after having started or completed our review. Readers may disagree with our approach to assess the risk of bias of individual studies, but we feel we provide enough information for readers to be able to assign weight to their preferred risk of bias element.

P.13, paragraph 2. What you have included is fine but I think you also need to explicitly allow for another possible scenario - the fact that the lack of evidence might simply be because of the methodological challenges of doing studies in this area that don’t suffer from some methodological limitation.

P.13, paragraph 2 does not seem to match the above comment. That said, we agree with this comment and it’s fairly similar to what we tried to convey at the beginning of the discussion section.

“It is important to note that we did not find evidence of no effect. Rather, we conclude that the evidence in LMICs was too limited and methodologically weak to make any conclusive statements.”
